# Alteration in Metabolic Signature and Lipid Metabolism in Patients with Angina Pectoris and Myocardial Infarction

**DOI:** 10.1371/journal.pone.0135228

**Published:** 2015-08-10

**Authors:** Ju Yeon Park, Sang-Hak Lee, Min-Jeong Shin, Geum-Sook Hwang

**Affiliations:** 1 Integrated Metabolomics Research Group, Western Seoul Center, Korea Basic Science Institute, Seoul, Republic of Korea; 2 Cardiology Division, Department of Internal Medicine, Yonsei University College of Medicine, Seoul, Republic of Korea; 3 Department of Public Health Sciences, Graduate School, Korea University, Seoul, Republic of Korea; 4 Department of Life Science, Ewha Womans University, Seoul, Republic of Korea; Mayo Clinic, UNITED STATES

## Abstract

Lipid metabolites are indispensable regulators of physiological and pathological processes, including atherosclerosis and coronary artery disease (CAD). However, the complex changes in lipid metabolites and metabolism that occur in patients with these conditions are incompletely understood. We performed lipid profiling to identify alterations in lipid metabolism in patients with angina and myocardial infarction (MI). Global lipid profiling was applied to serum samples from patients with CAD (angina and MI) and age-, sex-, and body mass index-matched healthy subjects using ultra-performance liquid chromatography/quadruple time-of-flight mass spectrometry and multivariate statistical analysis. A multivariate analysis showed a clear separation between the patients with CAD and normal controls. Lysophosphatidylcholine (lysoPC) and lysophosphatidylethanolamine (lysoPE) species containing unsaturated fatty acids and free fatty acids were associated with an increased risk of CAD, whereas species of lysoPC and lyso-alkyl PC containing saturated fatty acids were associated with a decreased risk. Additionally, PC species containing palmitic acid, diacylglycerol, sphingomyelin, and ceramide were associated with an increased risk of MI, whereas PE-plasmalogen and phosphatidylinositol species were associated with a decreased risk. In MI patients, we found strong positive correlation between lipid metabolites related to the sphingolipid pathway, sphingomyelin, and ceramide and acute inflammatory markers (high-sensitivity C-reactive protein). The results of this study demonstrate altered signatures in lipid metabolism in patients with angina or MI. Lipidomic profiling could provide the information to identity the specific lipid metabolites under the presence of disturbed metabolic pathways in patients with CAD.

## Introduction

Lipids are essential regulators of biological processes associated with normal cell function, metabolism, and distribution. Changes in lipid components secondary to genetic alterations, environmental influences, or both can have profound effects on cell function, the immune system, and inflammatory responses [1,2]. These effects can cause various lipid dysregulation-related diseases, including obesity [3], diabetes mellitus [4], and coronary artery disease (CAD) [5].

CAD patients exhibit an increased production of reactive oxygen species and compromised endogenous anti-oxidant defenses [6]. Previous studies demonstrated that endothelial dysfunction and increased oxidative stress are associated with the dysfunction and dysregulation of individual lipids. Abnormal lipid profiles stimulate endothelial activation, which upregulates adhesion molecules and promotes monocyte adhesion [7,8]. Furthermore, the overexposure of endothelial cells to lipids can amplify inflammatory response-mediated oxidative stress [9]. Impaired endothelial function has been linked to increased oxidative stress and altered lipid metabolism [10]. Observational studies reported that the arterial stiffness correlated positively with specific lipid and oxidative stress. In addition, lipid oxidation metabolism is associated with oxidized low-density lipoprotein (LDL) production and inflammation. Therefore it is necessary to investigate the complex changes involved in lipid metabolism in CAD patients.

The advent of analytical chemistry and information technology has made it feasible to measure large numbers of metabolites in biofluids and tissues. The focus of recent studies has tended to shift from determining the individual characteristics of lipids in biosamples to characterizing global changes in lipid metabolites in an integrated context to understand the role of lipids in pathophysiology. This line of study is known as lipidomics [11]. Lipidomics is the systems-based study of all lipids, the molecules with which they interact, and their functions within the cell. Lipid profiles, the composition and abundance of crude extracted lipids, contribute to our understanding of changes in individual lipids, lipid metabolism, and lipid oxidation. Therefore lipidomics could be a powerful tool for elucidating the mechanisms of lipid-based diseases, biomarker discovery, and monitoring therapeutic efficacy [12]. Lipidomic approaches have been applied to investigate obesity [13], diabetes [14], and vascular diseases [15,16] to characterize global lipid profiles and identify unknown changes in lipid metabolism.

A recent study showed changes in the aorta and plasma lipidome in a diet-induced mouse model of early atherogenesis [15], suggesting that the increase in glycerophospholipids and sphingolipids could be explained by the increased LDL cholesterol concentration, leading to a high risk of vascular disease. Observational studies have shown that the potential of plasma lipid profiling for the identification of CAD [16]. It was reported that plasma lipid profiling might have diagnostic and prognostic potential for the identification of patients at risk for unstable CAD.

Despite such efforts, the metabolic signatures of CAD, angina pectoris, and myocardial infarction (MI) have not been clearly elucidated. In the present study, we performed global lipid profiling to identify the most commonly altered serum lipid metabolites in patients with CAD using ultra-performance liquid chromatography/quadruple time-of-flight mass spectrometry (UPLC/Q-TOF MS). We also investigated the lipidomic signatures of patients with MI and compared them with those of healthy controls and patients with angina to understand the distinct mechanisms leading to MI.

## Materials and Methods

### Subjects

This study included 140 patients with CAD and 70 control subjects without CAD. The study subjects were drawn from the database of the Cardiovascular Genome Center at Severance Hospital in Seoul, Korea. Patients with CAD, including angina pectoris and MI, were included if they underwent coronary angiography for either chest discomfort or chest pain; had angiographically confirmed CAD with 50% stenosis of one or more epicardial coronary arteries; were not diagnosed with diabetes, cancer, renal disease, liver disease, thyroid disease, or acute or chronic inflammatory disease; or had MI confirmed according to World Health Organization’s criteria for symptoms including chest enzyme elevation, or electrocardiographic changes [17]. MI patients in the acute stage were included, and the time since MI onset was 1.97±2.67 days; blood samples were collected from all patients. All MI patients with chest symptoms underwent coronary angiography and intervention and did not have post-MI angina. Patients were classified to have unstable angina when they are with 1) angina pectoris occurs at rest or with minimal exertion, 2) severe and newly developed angina, and/or 3) angina with crescendo pattern. Stable angina was defined as chest pain or discomfort reproducibly associated with physical exertion and relieved by rest. All patients with unstable or stable angina were angiographically proven or with objective evidence of myocardial ischemia on stress tests. As a result, our study included a total of 70 angina patients composed of 55 stable angina patients and 15 unstable angina patients. A total of 70 age- and sex-matched (within a 2-year difference) healthy control participants were recruited from the Health Promotion Center at Severance Hospital. The exclusion criteria were a diagnosis of vascular disease, diabetes, cancer, renal disease, liver disease, thyroid disease, or acute or chronic inflammatory disease, or the current administration of lipid- or glucose-lowering medications.

Written informed consent was obtained from all participants, and the protocol was approved by the institutional review board of Severance Hospital.

### Anthropometric parameters and blood collection

Body weights and heights were measured in the morning for all subjects (unclothed) to calculate the body mass index (BMI) (kg/m^2^). Blood pressure was measured in the left arm of seated participants with an automatic blood pressure monitor (A&D, Tokyo, Japan) after a 20-min rest. After a 12-h fasting period, venous blood specimens were collected in plain tubes, centrifuged to yield serum, and stored at -70°C until analysis.

### Measurement of serum lipid profiles and fasting glucose

Fasting serum total cholesterol, triglyceride, and high-density lipoprotein (HDL) cholesterol levels were measured using commercially available kits on a Hitachi 7150 Autoanalyzer (Hitachi Ltd., Tokyo, Japan). Low-density lipoprotein (LDL) cholesterol was indirectly estimated in subjects with serum triglyceride concentrations of <400 mg/dl using the Friedewald formula. Fasting glucose levels were measured using the glucose oxidase method with a Beckman Glucose Analyzer (Beckman Instruments, Irvine, CA).

### Lipid metabolite analysis

Serum samples were thawed to room temperature, and extraction was performed using a chloroform:methanol mixture (2:1, v/v) as a modified method by Folch et al [18]. Serum lipid extracts were diluted with an isopropanol:acetonitrile:water mixture (2:1:1, v/v/v), and injection was used for UPLC/Q-TOF MS. LC-MS was performed on a triple TOF 5600 MS/MS system (AB SCIEX, Concord, Canada) combined with a UPLC system (Waters, Milford, MA). Separations were performed on an Acquity UPLC BEH C18 column (2.1 × 100 mm with 1.7 μm particles; Waters). The binary gradient system comprised 10 mM ammonium acetate in an acetonitrile:water (4:6, v/v) and 10 mM ammonium acetate in an acetonitrile:isopropanol (1:9, v/v). The flow rate was maintained at 0.35 ml/min for 16 min. The mass spectrometer was operated in the electrospray ionization-positive and -negative modes, and the mass range was set at m/z 100–1500. Mass accuracy was maintained with an automated calibrant delivery system interfaced to the second inlet of the DuoSpray source.

All MS data, including retention times, m/z, and ion intensities, were extracted by the Markerview software (AB Sciex, Concord, Canada) incorporated within the instrument, and the resulting data were assembled into a matrix. Metabolites were identified using the LipidMap (www.lipidmaps.org) and Human Metabolome databases (www.hmdb.ca) and confirmed using standard samples (Avanti Polar Lipids, Alabaster, AL) based on retention time, mass spectra, and precursor ion.

### Serum high-sensitivity C-reactive protein (hs-CRP) concentration

Serum hs-CRP concentrations were measured using an Express plus Autoanalyzer (Chiron Diagnostics Co., Walpole, MA) with a commercially available, hs-CRP-Latex (II) X2 kit (Seiken Laboratories Ltd., Tokyo, Japan).

### Statistical analysis

A statistical analysis was performed with Statistical Package for the Social Sciences software, version 15.0 (SPSS 15.0; SPSS Inc., Chicago, IL). False discovery rate (FDR) analysis was conducted using the q-value program in R version 2.15.3. All results are expressed as the means ± standard errors (SE) or proportions (%). The Differences in clinical characteristics were tested by one-way analysis of variance (ANOVA) with bonferroni’s multiple comparisons test and a two-tailed p-value <0.05 was considered to be statistically significant. After adjustment for age, sex, BMI, LDL cholesterol, and fasting glucose, differences in serum lipid metabolites between patients with cardiovascular disease (CAD) and control subjects were calculated using general linear model controlling the false discovery rate (FDR) and q-value <0.05 were considered to be statistically significant. The risk of angina or MI by alteration of specific lipid metabolites was calculated using a logistic regression model after adjusting for age, sex, BMI, LDL cholesterol, and fasting glucose, and was presented as the odds ratios (ORs) and 95% confidence intervals (CIs). Relationships between specific lipid metabolites and acute inflammatory responses were evaluated using pearson and partial correlation coefficients. Partial correlation coefficients was calculated after controlling for age, sex, BMI, LDL cholesterol, and fasting glucose, and q-value <0.05 were considered to be statistically significant.

A multivariate pattern analysis was performed using SIMCA-P+ software, version 12.0 (Umetrics, Umea, Sweden). Cross-validation with seven cross-validation groups was used throughout the analysis to determine the number of principal components. A partial least-squares discriminant analysis (PLS-DA) and orthogonal partial least-squares discriminant analysis (OPLS-DA) were used as classification methods to model the discrimination by visualizing the score plot. The projection (VIP) score was calculated based on the PLS weights, and the variability was explained in the PLS-DA. Metabolites with a VIP of >1 were considered to be the most important metabolites responsible for the differentiation of patients with angina or MI and control subjects.

## Results

### Anthropometric and biochemical characteristics

The anthropometric and biochemical characteristics of the controls and patients with CAD (angina pectoris and MI) are shown in Table 1. The patients with CAD had higher serum concentrations of fasting glucose and lower concentrations of HDL cholesterol and triglycerides than did the controls. The serum concentrations of total cholesterol and LDL cholesterol were significantly different between the patients with angina and the controls. Drug treatments were not significantly different between the patients with angina and those with MI, with the exception of ACE inhibitors and calcium antagonists (S1 Table). When we subdivided the patients with CAD into two groups according to treatment with lipid-lowering drugs (LLD), those not treated with LLD showed significantly higher serum concentrations of LDL cholesterol than did those treated with LLD in both the angina and MI groups (S2 Table).

**Table 1:** General characteristics of the controls and patients with coronary artery disease.

	Controls (n = 70)	Angina (n = 70)	MI ^2^ (n = 70)	P-value ^4^
Age (years)	63.4 ± 1.1	64.9± 1.0	65.7± 1.3	0.350
Males/Females (n)	36/34	36/34	36/34	
Body mass index (kg/m^2^)	23.1 ± 0.3	23.7± 0.3	23.3± 0.5	0.631
Blood pressure (mmHg)				
Systolic	127.0± 2.1	124.6± 1.5	122.3± 2.9	0.320
Diastolic	78.2± 1.1	77.5± 1.2	74.6± 1.9	0.154
Fasting glucose (mg/dl) ^**1**^	90.0± 2.0 ^**c**^	109.6± 2.5 ^**b**^	128.7± 3.0 ^**a**^	<0.001
Total cholesterol (mg/dl)	192.9± 3.9 ^**a**^	164.0± 5.4 ^**b**^	195.0± 4.6 ^**a**^	<0.001
Triglycerides (mg/dl) ^**1**^	142.0± 9.2 ^**a**^	113.0± 9.7 ^**b**^	111.9± 6.1 ^**b**^	0.009
HDL cholesterol (mg/dl) ^**1**^	51.6± 1.7 ^**a**^	42.8± 1.3 ^**b**^	43.9± 1.6 ^**b**^	<0.001
LDL cholesterol (mg/dl) ^**1**^	114.0± 3.7 ^**a**^	99.1± 4.6 ^**b**^	129.2± 4.7 ^**a**^	<0.001
Diabetes category (n) ^**3**^				
Impaired fasting glucose	7	26	29	<0.001
Diabetes	3	15	34	

The data are presented as the mean ± SE.

^1^Tested by log-transformed.

^2^ MI: myocardial infarction.

^3^ Diabetes category: Impaired fasting glucose, 100≤ fasting glucose <126mg/dL; Diabetes, fasting glucose ≥126mg/dL.

^4^ Tested by one-way analysis of variance (ANOVA) with bonferroni’s multiple comparisons test (p<0.05).

Sharing the same alphabet indicates no significant difference between two groups.

### Global pattern analysis of lipid metabolites

**Fig 1:**
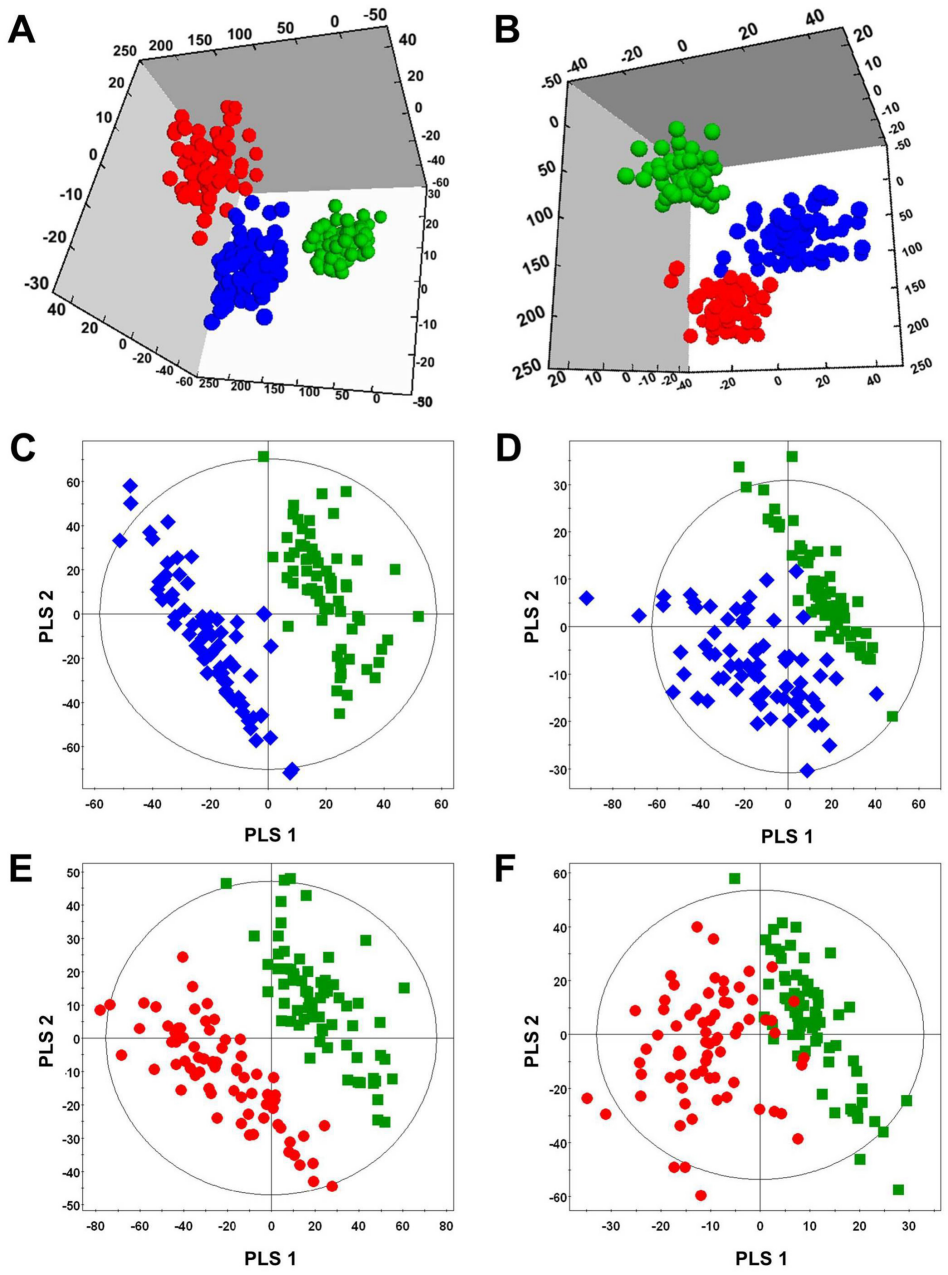
OPLS-DA and PLS-DA score plots. OPLS-DA score plots from the spectra of the positive (A) and negative (B) mode of UPLC/Q-TOF MS in serum lipid metabolites of controls (green) and patients with angina (blue) and MI (red). PLS-DA score plots from the spectra of the positive (C and E) and negative (D and F) mode of UPLC/Q-TOF MS.

We obtained a total of 3591 and 1725 aligned spectral features in UPLC/Q-TOF positive and negative modes, respectively, from CAD patients and control subjects; PLS-DA and OPLS-DA were performed to discriminate between patients with CAD and controls. An OPLS-DA model (one predictive component and two orthogonal components) was constructed to investigate the difference between patients with angina and MI in comparison with control subjects (Fig 1A and 1D). The cumulative R^2^Y and Q^2^ values were 83.0 and 71.5% in the positive mode and 68.0 and 59.4% in the negative mode, respectively.

The PLS-DA model showed a clear separation between the patients with angina and control subjects in both the positive mode (R^2^Y = 97.0, Q^2^ = 92.6) (Fig 1B) and negative mode (R^2^Y = 94.1, Q^2^ = 84.4) (Fig 1E), and between patients with MI and control subjects in both the positive mode (R^2^Y = 92.4, Q^2^ 85.5) (Fig 1C) and negative mode (R^2^Y = 93.9, Q^2^ = 81.6) (Fig 1F).

In additional, PLS-DA was conducted to observe the metabolic pattern between patients with stable and unstable angina in both positive and negative modes (S1 Fig).

### Altered lipid metabolites in patients with CAD

We identified 104 lipid metabolites in the positive mode and 54 lipid metabolites in the negative mode (S3 and S4 Tables). To investigate lipid metabolites associated with CAD, the following selection criteria were used: (a) a VIP score of >1 in the PLS-DA and (b) a general linear model after adjustment for age, sex, BMI, LDL cholesterol, and fasting glucose between patients with angina or MI and controls (q<0.05). Among the annotated lipid metabolites, free fatty acids (FFA), lysophosphatidylcholine (lysoPC), and lysophosphatidylethanolamine (lysoPE) were identified as candidate markers shared by patients with angina and MI compared with the controls.

**Fig 2:**
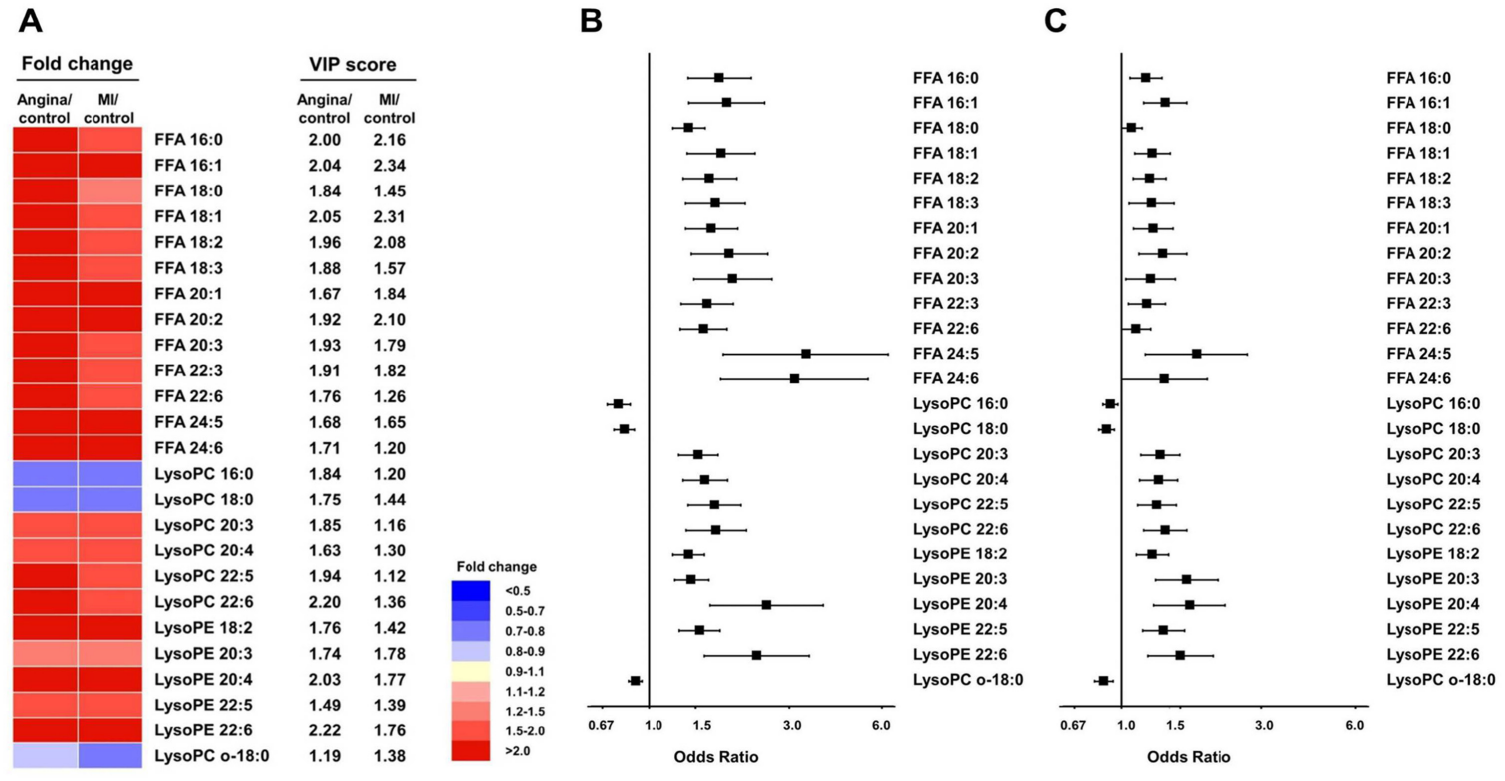
Commonly altered lipid metabolites in patients with CAD. Heat map showing the fold changes in and VIP scores of commonly altered metabolites in patients with CAD compared with control subjects (A) and the risk of angina (B) and MI (C). The values are ORs (95% CIs) increment for angina or MI from the logistic regression. All models are adjusted for age, sex, BMI, LDL cholesterol, and fasting glucose.

The levels of species of FFA, lysoPCs containing unsaturated fatty acids, and lysoPEs containing unsaturated fatty acids were higher in patients with CAD than in control subjects, whereas the levels of species of lysoPC and lyso-alkyl PC containing saturated fatty acids (SFA) were lower in patients with CAD (Fig 2A and S5 Table). Particularly, the levels of FFAs and lysoPCs containing unsaturated fatty acids were significantly higher in angina patients than MI patients. Stable and unstable angina showed significantly higher levels of FFA species compared to MI, whereas difference in FFA between stable and unstable angina was not observed (S6 Table).

We fit logistic regression models to assess the risk of angina and MI after adjustment for age, sex, BMI, LDL cholesterol, and fasting glucose (Fig 2B and 2C and S7 Table). For the 22 lipid metabolites, including lysoPC, lysoPE, and FFA, the markers were associated with a 1.09–3.84-fold increased risk of angina and MI. For the three negative predictors, including lysoPC-containing saturated long-chain fatty acids, the markers were associated with a 0.77–0.91-fold decreased risk of angina and MI.

### Altered lipid metabolites in patients with MI

To investigate whether specific lipid metabolites were associated with MI, the following metabolite selection criteria were used: (a) a VIP score of >1 in the PLS-DA and (b) a general linear model after adjustment for age, sex, BMI, LDL cholesterol, and fasting glucose between patients with MI and controls and between patients with MI and those with angina (q < 0.05). Among the annotated lipid metabolites, PC, phosphatidylinositol (PI), PE-plasmalogens. diacylglycerol (DG), sphingomyelin (SM), ceramide (Cer), and glucosylceramide (Glucer) were identified as specific markers of MI compared with control and angina.

**Fig 3:**
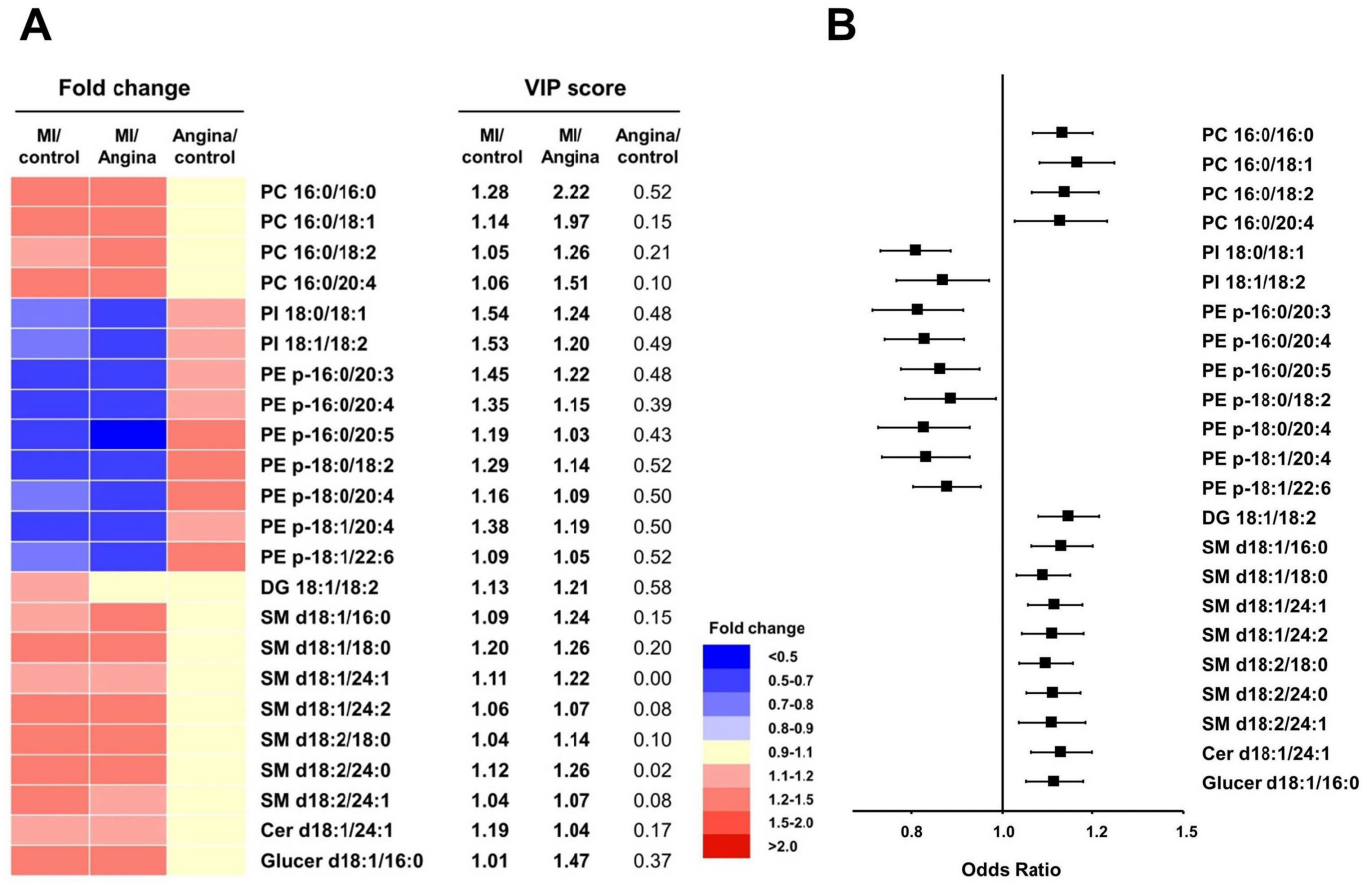
Significantly altered lipid metabolite in patients with MI. Heat map showing the fold changes in and VIP scores of altered metabolites in patients with MI compared with control subjects and patients with angina (A) and the risk of MI (B). The values are ORs (95% CIs) increment for MI from the logistic regression. All models are adjusted for age, sex, BMI, LDL cholesterol, and fasting glucose.

The levels of PC, DG, SM, Cer, and Glucer were higher in the patients with MI compared with control subjects, whereas the levels of PE-plasmalogens and PI were lower in the patients with MI (Fig 3A and S5 Table).

We fit logistic regression models to assess the risk of MI after adjustment for age, sex, BMI, LDL cholesterol, and fasting glucose (Fig 3B and S7 Table). For the 14 lipid metabolites associated with an increased risk of MI, including PC, DG, SM, Cer, and Glucer, these markers were associated with a 1.11–1.17-fold increased risk of MI. For the nine negative predictors, including PE-plasmalogen and PI, the markers were associated with a 0.83–0.89-fold decreased risk of MI.

### Association of specific metabolites of lipid metabolism and acute inflammation markers

**Fig 4:**
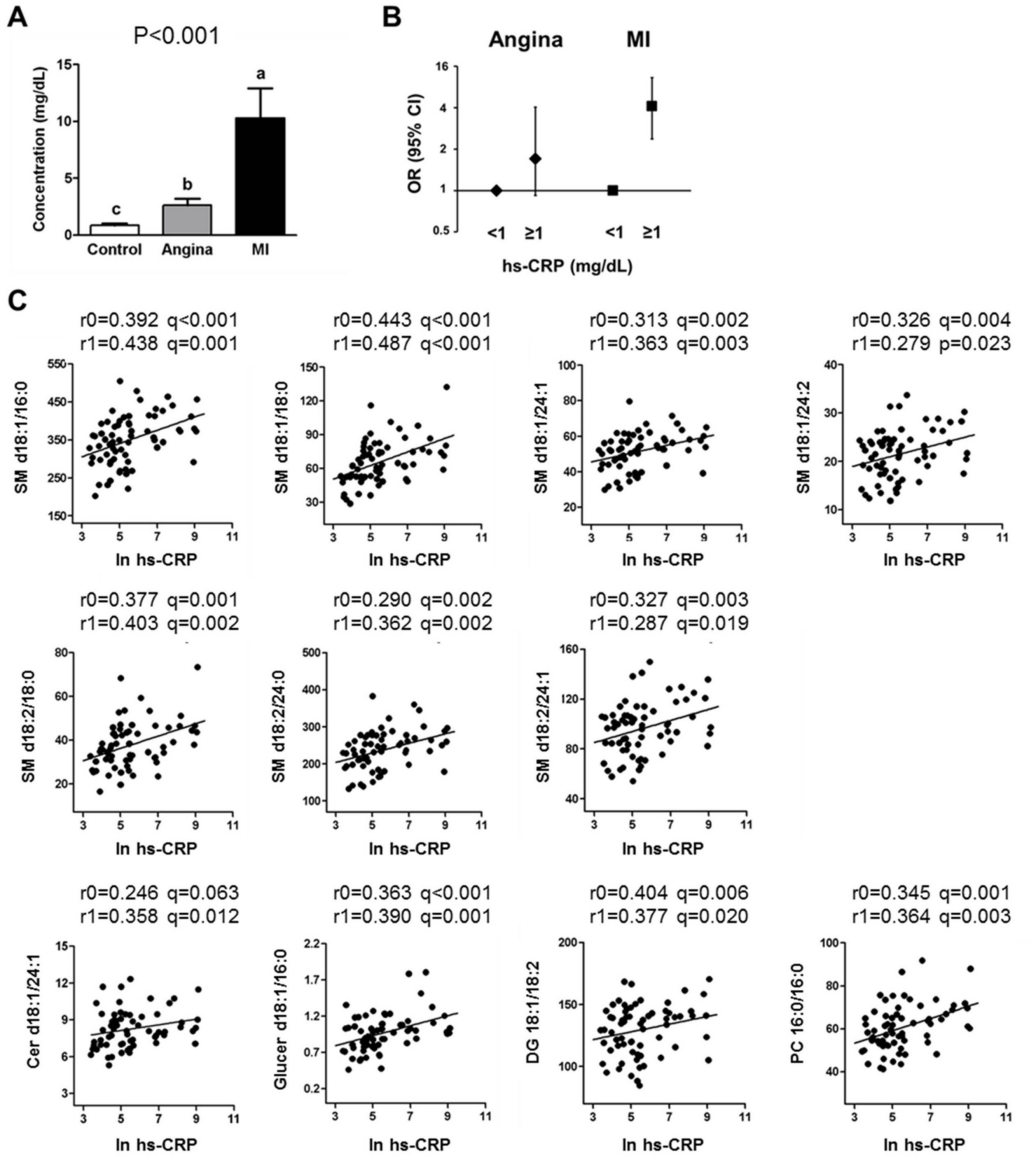
Association of specific metabolites and the acute inflammatory response. (A) Serum hs-CRP concentration of the controls and patients with CAD (A) as tested by a one-way ANOVA with the Bonferroni post-hoc test. Groups with different superscript letters were significantly different (P < 0.05). Risk of CAD according to the hs-CRP concentration (B). The values are ORs (95% CIs) from the logistic regression. All models are adjusted for age, sex, BMI, LDL cholesterol, and fasting glucose. Significant associations between specific metabolites of sphingolipid metabolism and acute inflammation markers (C). Pearson’s (r0) and partial correlation (r1) coefficients. r0: unadjusted; r1: adjusted for age, sex, BMI, LDL cholesterol, and fasting glucose.

The serum hs-CRP concentration was significantly higher in patients with angina and MI than in the control subjects (p < 0.001 and q < 0.001) (Fig 4A). When we divided the subjects according to the hs-CRP concentration, those with an hs-CRP concentration of ≥1 mg/dL had a 6.45-fold higher risk of MI after adjustment for confounding factors (Fig 4B).

Eleven lipid metabolites related to the sphingolipid pathway (SM, Cer, Glucer, DG, and PC) showed a positive correlation with hs-CRP before and after adjustment for confounding factors in patients with MI (Fig 4C and S8 Table). However, no significant correlation was found between the control and angina groups.

### Effect of LLD treatment on serum lipid metabolites

To examine the effect of LLD treatment on the levels of serum lipid metabolites, we performed a subanalysis according to treatment with LLD (S9 and S10 Tables). Eight species of lipid metabolites differed between the angina and control groups and three species between the MI and control groups. The p-values for these significant differences were maintained after adjustment for age, sex, BMI, LDL cholesterol, and fasting glucose (p < 0.05), but the q-values were not (q > 0.05). Despite the trend toward a decreased risk of angina or MI after LLD treatment, most lipid metabolites identified in the current study were unchanged by LLD in patients with angina or MI.

## Discussion

Altered lipid metabolism associated with inflammation and oxidative stress is one of the primary drivers of the pathological changes associated with atherosclerotic plaque formation [8,9]. Thus, to understand the complex alterations in lipid metabolism in patients with CAD, we performed global lipid profiling in serum using UPLC/Q-TOF MS. We also investigated the specific lipidomic signatures in patients with angina or MI to understand the different underlying mechanisms causing CAD.

Pattern recognition analysis indicated that this UPLC/Q-TOF MS-based lipidomics approach is capable of investigating the lipidomic differences between patients with CAD and control subjects. Furthermore, our study confirmed the presence of specific lipid metabolites and disturbed metabolic pathways by comparing patients with CAD and healthy controls. We found that FFA, lysoPC, and lysoPE may be strongly associated with CAD, particularly angina. We also confirmed that alterations in SM, Cer, Glucer, PC, PI, and PE-plasmalogens are associated with the risk of MI. These approaches are useful for the identification of important lipid metabolites that may play significant roles in the development of angina or MI.

Circulating lysoPC constitutes approximately 1 to 5% of the total PC content of LDL particles. However, as much as 40 to 50% of the PC in LDL particles is converted to lysoPC and FFA during LDL oxidation by PLA2, including secretory PLA2 and lipoprotein-associated phospholipase A2 or lecithin-cholesterol acyltransferase [19,20]. Circulating lysoPC has pro-atherogenic roles in monocyte recruitment, macrophage proliferation, smooth muscle cell proliferation, increased expression of endothelial adhesion molecules, and endothelial dysfunction [18–24]. For these reasons, lysoPC has been recognized as an independent risk factor for atherosclerosis [18–24]. In our study, patients with CAD showed higher levels of lysoPCs containing unsaturated fatty acids than did the control group. Similar to these significant increases, the serum levels and compositions of unsaturated FFA were also increased in patients with CAD. In addition, Levels of FFA and lysoPCs were higher in patients with angina than MI. These alterations in lysoPCs and FFA might be partly due to increased production from PLA2-catalyzed PC hydrolysis. This hypothesis is consistent with a previous report in which high levels of lysoPC during oxidative modification were found in patients with atherosclerosis and CAD [25,26]. Moreover, upregulated synthesis and an abnormal composition of lipids contributed to oxidative stress/inflammation, subsequent platelet aggregation, and vessel thrombosis in previous studies [5,27].

Interestingly, our study showed that lysoPCs containing unsaturated fatty acids were down-regulated in patients with CAD, whereas lysoPCs containing SFA were upregulated in these patients. The signaling properties and biological effects of lysoPC in association with atherosclerosis have been observed in previous studies. LysoPC activates a wide range of cell types in the vascular system and is known to have either pro- or anti-atherogenic effects depending on the cell type or oxidation/inflammation status [21–24,28]. Our study suggests that the function of lysoPC is affected by the cell type or oxidation/inflammation status, as well as the species of combined fatty acids. Similar to our results, a previous study showed that lysoPC 20:3, lysoPC 20:4, and lysoPC 22:6 are positively associated with CAD, whereas species of lysoPC containing SFA are negatively associated with CAD [16].

We found opposing alterations in PC and PE-plasmalogens, which are glycerophospholipid metabolism-related lipid metabolites, in patients with MI compared with healthy subjects and patients with angina. These inverse characteristics of PC and PE-plasmalogen in patients with CAD have been demonstrated in previous studies. Increased free fatty acid levels and the secretion of very low density lipoprotein and LDL promote the biosynthesis of PC, which is regulated by activation of the CDP-choline pathway [29,30]. Additionally, the activation of PC synthesis may lead to an increase in SM and the accumulation of pro-apoptotic Cer. PC and sphingolipids are involved in cell proliferation, differentiation, and death [31]. Therefore, PC has been recognized as an independent risk factor for atherosclerosis [29–31]. On the other hand, plasmalogens, especially PE-plasmalogen, act as antioxidants and protect endothelial cells from oxidative injury. Plasmalogens are able to protect unsaturated membrane lipids against oxidation by reactive oxygen species without producing excessive toxic oxidation products [32]. Our study is consistent with previous reports; altered levels of PC and PE in patients with MI might be the result of the different mechanism between MI and angina (e.g., cell apoptosis and oxidative stress).

We also observed that the upregulation of sphingolipids is associated with an increased risk of MI. The sphingolipid, SM, and Cer signaling pathway is altered by stress, including oxidative stress, and is critically involved in cell proliferation and death as well as the contraction of cardiomyocytes and vascular smooth muscle cells [31,33]. Various stressful conditions stimulate the catabolism of SM by sphingomyelinase [34]. The results of the present study might be due to thrombus formation and the development of MI. In line with our results, previous observations have shown increased sphingolipid levels and activated sphingomyelinase activity during atherogenesis [15].

Sphingolipids are a family of lipid second messengers that regulate various vascular cell functions. In particular, SM and Cer mediate cellular responses to cytokines and oxidative stress. The sphingolipid level is reportedly regulated during the acute phase of the response to inflammation, and increases in Cer and sphingosine lead to the upregulation of different acute-phase proteins [35]. Moreover, elevation in the serum hs-CRP level has been suggested to be associated with an increased risk of CAD [36]. These findings are supported by our observation that the sphingolipid level was significantly associated with the hs-CRP level in patients with MI.

The limitations of our study include the use of LLD, which affects lipid metabolism, in patients with CAD. This was addressed in the analysis of CAD with LLD treatment as a covariate in the multivariable logistic analysis. Additionally, the subanalysis of LLD treatment status showed significance in only two species of CAD-related lipid metabolites, and these metabolites showed a relationship with CAD risk regardless of LLD.

Overall, we found that lysoPC and lysoPE species containing unsaturated fatty acids and FFAs were associated with an increased risk of CAD, whereas species of lysoPC and lyso-alkyl PC containing saturated fatty acids were associated with a decreased risk. Additionally, PC species containing palmitic acid, DG, SM, and Cer were associated with an increased risk of MI, whereas PE-plasmalogen and phosphatidylinositol species were associated with a decreased risk. Finally, global lipidomic profiling of patients with angina or MI suggested common alterations in lipid metabolism in these populations. We also confirmed different metabolic signatures resulting from acute inflammatory responses and upregulated sphingolipid and glycerophospholipid in patients with MI. In conclusion, lipidomic profiling was used successfully to identify specific metabolites in patients with angina or MI. In addition, specific metabolites could use to diagnose developing CAD at an early stage.

## Supporting Information

S1 Fig PLS-DA score plots of CAD patients. PLS-DA score plots from the spectra of the positive (left) and negative (right) mode of UPLC/Q-TOF MS in serum lipid metabolites of patients with stable angina, unstable anagina, and MI.
(DOCX)

S1 Table Drug treatment in patients with angina and MI. (DOCX)

S2 Table Serum glucose and cholesterol levels in patients with angina and MI according to lipid-lowering treatment. (DOCX)

S3 Table Identified lipid metabolites by class and number of significant association with angina and MI. (DOCX)

S4 Table Information of identified lipid metabolites. (DOCX)

S5 Table Levels of individual lipid species in patients with CAD and control subjects. (DOCX)

S6 Table Levels of individual lipid species in patients with CAD. (DOCX)

S7 Table Odds ratio for angina or MI of individual lipid species. (DOCX)

S8 Table Association of specific metabolites and the acute inflammatory response. (DOCX)

S9 Table Levels of individual lipid species in patients with angina and MI according to statin treatment. (DOCX)

S10 Table ORs for CAD of individual lipid species associated with statin treatment. (DOCX)
