# Supplementary material for: Alteration in Metabolic Signature and Lipid Metabolism in Patients with Angina Pectoris and Myocardial Infarction
Source: PLoS One. 2015 Aug 10;10(8):e0135228. doi: 10.1371/journal.pone.0135228 (PMC4530944; doi:10.1371/journal.pone.0135228)
Supplement: S1 Fig — PLS-DA score plots from the spectra of the positive (left) and negative (right) mode of UPLC/Q-TOF MS in serum lipid metabolites of patients with stable angina, unstable anagina, and MI. (DOCX) [file pone.0135228.s001.docx]

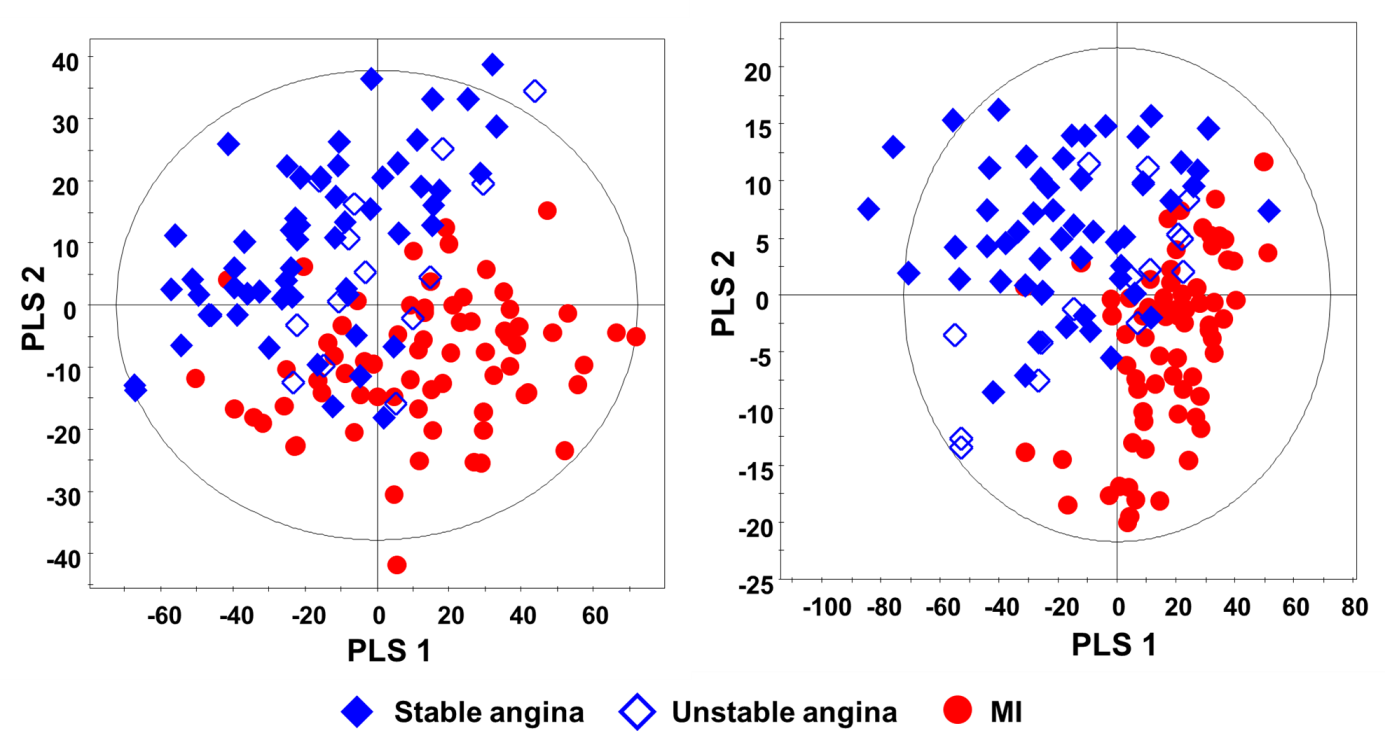


**S1 Fig. PLS-DA score plots of CAD patients.** PLS-DA score plots from the spectra of the positive (left) and negative (right) mode of UPLC/Q-TOF MS in serum lipid metabolites of patients with stable angina, unstable anagina, and MI.
