# Supplementary material for: Alteration in Metabolic Signature and Lipid Metabolism in Patients with Angina Pectoris and Myocardial Infarction
Source: PLoS One. 2015 Aug 10;10(8):e0135228. doi: 10.1371/journal.pone.0135228 (PMC4530944; doi:10.1371/journal.pone.0135228)
Supplement: S1 Table — (DOCX) [file pone.0135228.s002.docx]

**S1 Table. Drug treatment in patients with angina and MI**

| Medication | Angina (%) | MI (%) | P-value |
| --- | --- | --- | --- |
| Antiplatelet | 97.1 | 90.0 | 0.085 |
| Anticoagulant | 94.3 | 95.7 | 0.698 |
| Lipid-lowering | 62.9 | 62.9 | 1.000 |
| Vasodilator | 54.3 | 51.4 | 0.735 |
| ACE inhibitor | 7.1 | 61.4 | <0.001 |
| Alpha-blocker | 12.9 | 11.4 | 0.796 |
| Angiotensin-II receptor antagonist | 12.9 | 5.7 | 0.145 |
| Beta-blocker | 51.4 | 38.6 | 0.126 |
| Calcium antagonist | 32.9 | 11.4 | 0.002 |
| Diuretic | 8.6 | 18.6 | 0.084 |
