# Supplementary material for: Alteration in Metabolic Signature and Lipid Metabolism in Patients with Angina Pectoris and Myocardial Infarction
Source: PLoS One. 2015 Aug 10;10(8):e0135228. doi: 10.1371/journal.pone.0135228 (PMC4530944; doi:10.1371/journal.pone.0135228)
Supplement: S2 Table — (DOCX) [file pone.0135228.s003.docx]

**S2 Table. Serum glucose and cholesterol levels in patients with angina and MI according to lipid-lowering treatment**

|  | Angina | | | | |  | MI | | | | |
| --- | --- | --- | --- | --- | --- | --- | --- | --- | --- | --- | --- |
|  | Nontreatment | | Treatment | | P-value |  | Nontreatment | | Treatment | | P-value |
| Fasting glucose (mg/dl) ^§^ | 108.2 | ± 4.3 | 110.5 | ± 3.1 | 0.574 |  | 134.2 | ± 5.5 | 125.5 | ± 3.5 | 0.214 |
| Total cholesterol (mg/dl) | 184.9 | ± 8.6 | 151.0 | ± 6.2 | 0.002 |  | 203.8 | ± 8.3 | 189.8 | ± 5.5 | 0.146 |
| Triglycerides (mg/dl) ^§^ | 131.3 | ± 21.1 | 100.8 | ± 7.5 | 0.201 |  | 107.0 | ± 10.2 | 114.8 | ± 7.6 | 0.533 |
| HDL cholesterol (mg/dl) ^§^ | 43.6 | ± 1.7 | 42.2 | ± 1.8 | 0.444 |  | 41.7 | ± 2.1 | 45.2 | ± 2.3 | 0.398 |
| LDL cholesterol (mg/dl) ^§^ | 116.7 | ± 7.1 | 88.4 | ± 5.4 | **0.002** |  | 142.0 | ± 7.9 | 121.7 | ± 5.6 | **0.039** |

The data are presented as the mean ± SE. ^§^Tested by the log-transformed method.
