# Supplementary material for: Alteration in Metabolic Signature and Lipid Metabolism in Patients with Angina Pectoris and Myocardial Infarction
Source: PLoS One. 2015 Aug 10;10(8):e0135228. doi: 10.1371/journal.pone.0135228 (PMC4530944; doi:10.1371/journal.pone.0135228)
Supplement: S3 Table — (DOCX) [file pone.0135228.s004.docx]

**S3 Table. Identified lipid metabolites by class and number of significant association with angina and MI**

| Class | Abbreviation | Number of  Identified metabolites | | Number of  significant association | |
| --- | --- | --- | --- | --- | --- |
|  |  | Total (positive/negative mode) | | Angina vs  control | MI vs  control |
| Free fatty acid | FFA | 15 | (0/15) | 15 | 13 |
| Lysophosphatidylcholine | LysoPC | 15 | (15/9) | 11 | 9 |
| Lysophosphatidylethanolamine | LysoPE | 11 | (11/6) | 10 | 8 |
| Alkyllysophosphatidylcholine | LysoPC-o | 2 | (2/0) | 2 | 2 |
| Lysophosphatidylcholine-plasmalogen | LysoPC-p | 1 | (1/0) | 1 | 0 |
| Phosphatidylcholine | PC | 31 | (31/0) | 1 | 12 |
| Phosphatidylethanolamine | PE | 6 | (0/6) | 5 | 2 |
| Phosphatidylinositol | PI | 9 | (0/9) | 2 | 7 |
| Alkylphosphatidylcholine | PC-o | 8 | (8/0) | 0 | 3 |
| Phosphatidylcholine-plasmalogen | PC-p | 5 | (5/0) | 0 | 4 |
| Phosphatidylethanolamine-plasmalogen | PE-p | 9 | (0/9) | 5 | 9 |
| Diacylglycerol | DG | 3 | (3/0) | 2 | 2 |
| Sphingomyelin | SM | 17 | (17/0) | 0 | 14 |
| Ceramide | Cer | 3 | (3/0) | 1 | 3 |
| Glucosylceramide | Glucer | 2 | (2/0) | 0 | 1 |
| Cholesterylester | CE | 6 | (6/0) | 2 | 5 |

A two-tailed P-value of <0.05 and q value of <0.05 was considered statistically significant.
