# Supplementary material for: Alteration in Metabolic Signature and Lipid Metabolism in Patients with Angina Pectoris and Myocardial Infarction
Source: PLoS One. 2015 Aug 10;10(8):e0135228. doi: 10.1371/journal.pone.0135228 (PMC4530944; doi:10.1371/journal.pone.0135228)
Supplement: S4 Table — (DOCX) [file pone.0135228.s005.docx]

**S4 Table. Information of identified lipid metabolites**

|  | Retention time (min) | m/z | Adduct |
| --- | --- | --- | --- |
| FFA 16:0 | 3.41 | 255.2320 | M-H |
| FFA 16:1 | 2.52 | 253.2173 | M-H |
| FFA 18:0 | 4.69 | 283.2637 | M-H |
| FFA 18:1 | 3.62 | 281.2481 | M-H |
| FFA 18:2 | 2.82 | 279.2334 | M-H |
| FFA 18:3 | 2.21 | 277.2142 | M-H |
| FFA 20:1 | 4.91 | 309.2775 | M-H |
| FFA 20:2 | 3.87 | 307.2640 | M-H |
| FFA 20:3 | 3.16 | 305.2470 | M-H |
| FFA 20:4 | 2.67 | 303.2330 | M-H |
| FFA 20:5 | 2.10 | 301.2165 | M-H |
| FFA 22:3 | 4.23 | 333.2738 | M-H |
| FFA 22:6 | 2.41 | 327.2326 | M-H |
| FFA 24:5 | 3.83 | 357.2781 | M-H |
| FFA 24:6 | 3.26 | 355.2613 | M-H |
| LysoPC 14:0 | 2.17 / 2.02 | 468.3076 / 526.3102 | M+H / M+CH_3_COOH-H |
| LysoPC 16:0 | 3.02 / 2.99 | 496.3396 / 554.3403 | M+H / M+CH_3_COOH-H |
| LysoPC 16:1 | 2.34 | 494.3212 | M+H |
| LysoPC 18:0 | 3.53 / 4.17 | 524.3692 / 582.3776 | M+H / M+CH_3_COOH-H |
| LysoPC 18:1 | 3.10 / 3.31 | 522.3572 / 580.3626 | M+H / M+CH_3_COOH-H |
| LysoPC 18:2 | 2.58 / 2.42 | 520.3390 / 578.3448 | M+H / M+CH_3_COOH-H |
| LysoPC 18:3 | 2.04 | 518.3214 | M+H |
| LysoPC 18:4 | 1.63 | 516.3036 | M+H |
| LysoPC 20:1 | 3.55 | 550.3880 | M+H |
| LysoPC 20:3 | 2.84 / 2.73 | 546.3542 / 604.3668 | M+H / M+CH_3_COOH-H |
| LysoPC 20:4 | 2.49 / 2.32 | 544.3406 / 602.3431 | M+H / M+CH_3_COOH-H |
| LysoPC 20:5 | 1.98 | 542.3225 | M+H |
| LysoPC 22:5 | 2.63 / 2.46 | 570.3570 / 628.3619 | M+H / M+CH_3_COOH-H |
| LysoPC 22:6 | 2.36 / 2.20 | 568.3385 / 626.3442 | M+H / M+CH_3_COOH-H |
| LysoPC 24:0 | 5.45 | 608.4633 | M+H |
| LysoPE 16:0 | 3.09 / 3.13 | 454.2935 / 452.2766 | M+H / M-H |
| LysoPE 18:0 | 3.59 / 4.32 | 482.3236 / 480.3084 | M+H / M-H |
| LysoPE 18:1 | 3.17 / 3.29 | 480.3115 / 478.2918 | M+H / M-H |
| LysoPE 18:2 | 2.69 / 2.54 | 478.2938 / 476.2766 | M+H / M-H |
| LysoPE 18:3 | 2.09 | 476.2705 | M+H |
| LysoPE 20:1 | 3.06 | 508.3353 | M+H |
| LysoPE 20:3 | 2.92 | 504.3082 | M+H |
| LysoPE 20:4 | 2.58 / 2.43 | 502.2930 / 500.2761 | M+H / M-H |
| LysoPE 22:1 | 3.34 | 536.3714 | M+H |
| LysoPE 22:5 | 2.94 | 528.3101 | M+H |
| LysoPE 22:6 | 2.46 / 2.29 | 526.2920 / 524.2771 | M+H / M-H |
| LysoPC o-16:0 | 3.26 | 482.3586 | M+H |
| LysoPC o-18:0 | 3.29 | 510.3534 | M+H |
| LysoPC p-18:0 | 3.32 | 508.3747 | M+H |
| PC 14:0/18:2 | 5.84 | 730.5389 | M+H |
| PC 14:0/20:5 | 5.14 | 752.5239 | M+H |
| PC 16:0/16:0 | 7.59 | 734.5675 | M+H |
| PC 16:0/16:1 | 6.61 | 732.5541 | M+H |
| PC 16:0/18:0 | 8.74 | 762.6014 | M+H |
| PC 16:0/18:1 | 7.64 | 760.5864 | M+H |
| PC 16:0/18:2 | 6.80 | 758.5712 | M+H |
| PC 16:0/18:3 | 6.30 | 778.5330 | M+Na |
| PC 16:0/20:3 | 7.09 | 784.5859 | M+H |
| PC 16:0/20:4 | 6.65 | 782.5703 | M+H |
| PC 16:0/20:5 | 6.00 | 780.5541 | M+H |
| PC 16:0/22:4 | 7.42 | 810.6021 | M+H |
| PC 16:0/22:5 | 6.69 | 808.5879 | M+H |
| PC 16:0/22:6 | 6.39 | 806.5704 | M+H |
| PC 16:1/18:2 | 6.14 | 756.5553 | M+H |
| PC 16:1/20:4 | 5.50 | 780.5553 | M+H |
| PC 16:1/22:6 | 5.37 | 804.5555 | M+H |
| PC 18:0/18:1 | 8.80 | 788.6171 | M+H |
| PC 18:0/18:2 | 7.89 | 786.6011 | M+H |
| PC 18:0/20:3 | 8.16 | 812.6184 | M+H |
| PC 18:0/20:4 | 7.73 | 810.6000 | M+H |
| PC 18:0/20:5 | 7.00 | 808.5855 | M+H |
| PC 18:0/22:5 | 7.74 | 836.6188 | M+H |
| PC 18:0/22:6 | 7.43 | 834.6014 | M+H |
| PC 18:1/18:2 | 6.86 | 784.5853 | M+H |
| PC 18:1/22:6 | 6.44 | 832.5858 | M+H |
| PC 18:2/20:4 | 6.05 | 806.5709 | M+H |
| PC 20:0/18:2 | 8.91 | 814.6348 | M+H |
| PC 20:4/20:4 | 5.70 | 830.5704 | M+H |
| PC 20:4/22:6 | 5.53 | 854.5676 | M+H |
| PE 16:0/20:4 | 8.53 | 738.5089 | M-H |
| PE 18:0/18:1 | 8.53 | 744.5590 | M-H |
| PE 18:0/20:3 | 9.60 | 768.5554 | M-H |
| PE 18:0/20:4 | 9.27 | 766.5449 | M-H |
| PE 18:0/20:5 | 8.78 | 764.5283 | M-H |
| PE 18:1/18:2 | 8.69 | 740.5229 | M-H |
| PI 16:0/20:4 | 7.70 | 857.5214 | M-H |
| PI 18:0/18:1 | 8.85 | 863.5682 | M-H |
| PI 18:0/18:2 | 8.34 | 861.5515 | M-H |
| PI 18:0/20:3 | 8.50 | 887.5652 | M-H |
| PI 18:0/20:4 | 8.24 | 885.5515 | M-H |
| PI 18:0/22:5 | 8.27 | 911.5670 | M-H |
| PI 18:0/22:6 | 8.10 | 909.5514 | M-H |
| PI 18:1/18:2 | 7.80 | 859.5405 | M-H |
| PI 18:1/20:4 | 7.71 | 883.5274 | M-H |
| PC o-16:0/18:2 | 6.30 | 744.5528 | M+H |
| PC o-16:0/20:4 | 7.29 | 768.5921 | M+H |
| PC o-16:0/22:6 | 7.01 | 792.5878 | M+H |
| PC o-18:0/16:0 | 9.47 | 748.6221 | M+H |
| PC o-18:0/18:2 | 7.34 | 772.5845 | M+H |
| PC o-18:0/20:4 | 8.42 | 796.6217 | M+H |
| PC o-18:0/22:6 | 8.09 | 820.6212 | M+H |
| PC o-20:0/20:4 | 9.57 | 824.6529 | M+H |
| PC p-16:0/16:0 | 8.18 | 718.5689 | M+H |
| PC p-16:0/20:4 | 7.14 | 766.5769 | M+H |
| PC p-18:0/16:0 | 8.32 | 746.6077 | M+H |
| PC p-18:0/18:1 | 7.31 | 794.6044 | M+H |
| PC p-18:0/18:2 | 7.29 | 770.5954 | M+H |
| PE p-16:0/20:3 | 9.04 | 724.5312 | M-H |
| PE p-16:0/20:4 | 8.88 | 722.5172 | M-H |
| PE p-16:0/20:5 | 8.42 | 720.5020 | M-H |
| PE p-16:0/22:6 | 8.67 | 746.5109 | M-H |
| PE p-18:0/18:2 | 9.87 | 726.5372 | M-H |
| PE p-18:0/20:4 | 9.70 | 750.5448 | M-H |
| PE p-18:0/22:6 | 9.43 | 774.5417 | M-H |
| PE p-18:1/20:4 | 8.89 | 748.5280 | M-H |
| PE p-18:1/22:6 | 8.69 | 772.5300 | M-H |
| DG 16:0/18:1 | 8.37 | 612.5573 | M+NH4 |
| DG 18:1/18:1 | 8.39 | 638.5700 | M+NH4 |
| DG 18:1/18:2 | 8.12 | 636.5525 | M+NH4 |
| SM d16:1/23:0 | 9.42 | 773.6499 | M+H |
| SM d18:1/14:0 | 5.61 | 675.5425 | M+H |
| SM d18:1/16:0 | 6.59 | 703.5744 | M+H |
| SM d18:1/18:0 | 7.70 | 731.6056 | M+H |
| SM d18:1/20:0 | 8.85 | 759.6367 | M+H |
| SM d18:1/22:0 | 9.93 | 787.6683 | M+H |
| SM d18:1/23:0 | 10.29 | 801.6832 | M+H |
| SM d18:1/24:0 | 10.58 | 815.7007 | M+H |
| SM d18:1/24:1 | 9.84 | 835.6693 | M+Na |
| SM d18:1/24:2 | 8.84 | 833.6493 | M+Na |
| SM d18:2/16:0 | 5.71 | 701.5607 | M+H |
| SM d18:2/18:0 | 6.70 | 729.5884 | M+H |
| SM d18:2/20:0 | 7.79 | 757.6224 | M+H |
| SM d18:2/22:0 | 8.93 | 785.6541 | M+H |
| SM d18:2/23:0 | 9.51 | 799.6696 | M+H |
| SM d18:2/24:0 | 9.83 | 813.6855 | M+H |
| SM d18:2/24:1 | 8.84 | 811.6663 | M+H |
| Cer d18:0/24:1 | 11.05 | 650.6454 | M+H |
| Cer d18:1/22:1 | 10.80 | 620.5890 | M+H |
| Cer d18:1/24:1 | 10.52 | 648.6288 | M+H |
| Glucer d18:1/16:0 | 6.90 | 700.5782 | M+H |
| Glucer d18:1/22:0 | 10.12 | 784.6652 | M+H |
| CE 18:2 | 12.14 | 666.6160 | M+NH4 |
| CE 18:3 | 11.93 | 664.6069 | M+NH4 |
| CE 20:3 | 12.16 | 692.6369 | M+NH4 |
| CE 20:4 | 12.01 | 690.6154 | M+NH4 |
| CE 20:5 | 11.84 | 671.5752 | M+NH4 |
| CE 22:6 | 11.89 | 714.6204 | M+NH4 |
