# Supplementary material for: Alteration in Metabolic Signature and Lipid Metabolism in Patients with Angina Pectoris and Myocardial Infarction
Source: PLoS One. 2015 Aug 10;10(8):e0135228. doi: 10.1371/journal.pone.0135228 (PMC4530944; doi:10.1371/journal.pone.0135228)
Supplement: S5 Table — (DOCX) [file pone.0135228.s006.docx]

**S5 Table. Levels of individual lipid species in patients with CAD and control subjects**

| Class | Metabolites | Control | | | Angina | | | MI | | | Angina vs Control | | | MI vs Control | | | Angina vs MI | | |
| --- | --- | --- | --- | --- | --- | --- | --- | --- | --- | --- | --- | --- | --- | --- | --- | --- | --- | --- | --- |
|  |  |  |  |  |  |  |  |  |  |  | VIP | p value | FDR  q value | VIP | p value | FDR  q value | VIP | p value | FDR  q value |
| FFA | FFA 16:0 | 171.9 | ± | 6.7 | 471.5 | ± | 20.6 | 279.3 | ± | 12.2 | 2.00 | <0.001 | <0.001 | 2.16 | <0.001 | <0.001 | 1.42 | <0.001 | <0.001 |
|  | FFA 16:1 | 33.2 | ± | 1.7 | 132.1 | ± | 6.6 | 72.2 | ± | 4.2 | 2.04 | <0.001 | <0.001 | 2.34 | <0.001 | <0.001 | 1.37 | <0.001 | <0.001 |
|  | FFA 18:0 | 102.6 | ± | 4.0 | 215.1 | ± | 8.9 | 133.6 | ± | 5.3 | 1.84 | <0.001 | <0.001 | 1.45 | 0.022 | 0.008 | 1.40 | <0.001 | <0.001 |
|  | FFA 18:1 | 209.3 | ± | 8.8 | 664.4 | ± | 29.9 | 377.6 | ± | 17.7 | 2.05 | <0.001 | <0.001 | 2.31 | <0.001 | <0.001 | 1.44 | <0.001 | <0.001 |
|  | FFA 18:2 | 126.6 | ± | 6.3 | 425.9 | ± | 21.8 | 229.5 | ± | 12.6 | 1.96 | <0.001 | <0.001 | 2.08 | <0.001 | <0.001 | 1.39 | <0.001 | <0.001 |
|  | FFA 18:3 | 19.4 | ± | 1.3 | 94.0 | ± | 6.0 | 37.6 | ± | 3.3 | 1.88 | <0.001 | <0.001 | 1.57 | <0.001 | <0.001 | 1.44 | <0.001 | <0.001 |
|  | FFA 20:1 | 4.4 | ± | 0.3 | 16.9 | ± | 1.3 | 9.9 | ± | 0.8 | 1.67 | <0.001 | <0.001 | 1.84 | <0.001 | <0.001 | 0.92 | <0.001 | <0.001 |
|  | FFA 20:2 | 3.4 | ± | 0.2 | 14.7 | ± | 0.9 | 6.9 | ± | 0.4 | 1.92 | <0.001 | <0.001 | 2.10 | <0.001 | <0.001 | 1.40 | <0.001 | <0.001 |
|  | FFA 20:3 | 4.2 | ± | 0.2 | 18.8 | ± | 1.1 | 8.0 | ± | 0.6 | 1.93 | <0.001 | <0.001 | 1.79 | 0.001 | 0.001 | 1.46 | <0.001 | <0.001 |
|  | FFA 20:4 | 17.6 | ± | 0.9 | 45.8 | ± | 2.8 | 22.9 | ± | 1.3 | 1.67 | <0.001 | <0.001 | 1.14 | - | - | 1.34 | <0.001 | <0.001 |
|  | FFA 20:5 | 5.8 | ± | 0.5 | 24.9 | ± | 2.4 | 8.2 | ± | 0.9 | 1.45 | <0.001 | <0.001 | 0.79 | - | - | 1.21 | <0.001 | <0.001 |
|  | FFA 22:3 | 0.1 | ± | 0.0 | 0.4 | ± | 0.0 | 0.2 | ± | 0.0 | 1.91 | <0.001 | <0.001 | 1.82 | 0.001 | 0.001 | 1.37 | <0.001 | <0.001 |
|  | FFA 22:6 | 25.7 | ± | 1.8 | 110.0 | ± | 7.7 | 44.0 | ± | 4.3 | 1.76 | <0.001 | <0.001 | 1.26 | 0.016 | 0.006 | 1.35 | <0.001 | <0.001 |
|  | FFA 24:5 | 0.3 | ± | 0.0 | 3.3 | ± | 0.3 | 1.2 | ± | 0.2 | 1.68 | <0.001 | <0.001 | 1.65 | 0.001 | 0.001 | 1.14 | <0.001 | <0.001 |
|  | FFA 24:6 | 0.3 | ± | 0.0 | 2.2 | ± | 0.2 | 0.6 | ± | 0.1 | 1.71 | <0.001 | <0.001 | 1.20 | 0.007 | 0.003 | 1.33 | <0.001 | <0.001 |
| LysoPC | LysoPC 14:0 | 4.4 | ± | 0.2 | 3.1 | ± | 0.1 | 2.7 | ± | 0.1 | 1.56 | <0.001 | <0.001 | 1.64 | <0.001 | <0.001 | 0.62 | 0.004 | 0.002 |
|  | LysoPC 16:0 | 402.9 | ± | 9.6 | 296.7 | ± | 6.0 | 321.7 | ± | 10.0 | 1.84 | <0.001 | <0.001 | 1.20 | 0.001 | 0.001 | 0.61 | - | - |
|  | LysoPC 16:1 | 10.2 | ± | 0.3 | 9.3 | ± | 0.3 | 9.0 | ± | 0.3 | 0.54 | 0.049 | 0.030 | 0.61 |  | - | 0.34 | - | - |
|  | LysoPC 18:0 | 168.3 | ± | 4.4 | 122.3 | ± | 3.0 | 120.9 | ± | 4.5 | 1.75 | <0.001 | <0.001 | 1.44 | <0.001 | <0.001 | 0.46 | - | - |
|  | LysoPC 18:1 | 67.9 | ± | 2.6 | 71.8 | ± | 1.8 | 63.6 | ± | 2.3 | 0.31 | - | - | 0.28 |  | - | 1.09 | - | - |
|  | LysoPC 18:2 | 72.7 | ± | 2.7 | 116.5 | ± | 4.8 | 87.9 | ± | 5.1 | 1.65 | <0.001 | <0.001 | 0.59 | <0.001 | <0.001 | 1.89 | 0.014 | 0.007 |
|  | LysoPC 18:3 | 2.4 | ± | 0.1 | 3.2 | ± | 0.1 | 2.2 | ± | 0.1 | 1.02 | <0.001 | <0.001 | 0.21 |  | - | 1.91 | <0.001 | <0.001 |
|  | LysoPC 18:4 | 1.3 | ± | 0.0 | 1.3 | ± | 0.0 | 1.2 | ± | 0.0 | 0.06 | - | - | 0.37 |  | - | 0.37 | - | - |
|  | LysoPC 20:1 | 2.3 | ± | 0.1 | 2.1 | ± | 0.1 | 2.1 | ± | 0.1 | 0.31 | - | - | 0.34 | - | - | 0.80 | - | - |
|  | LysoPC 20:3 | 7.8 | ± | 0.2 | 15.2 | ± | 0.7 | 11.7 | ± | 0.6 | 1.85 | <0.001 | <0.001 | 1.16 | <0.001 | <0.001 | 1.34 | 0.015 | 0.007 |
|  | LysoPC 20:4 | 18.6 | ± | 0.5 | 31.8 | ± | 1.6 | 29.0 | ± | 1.5 | 1.63 | <0.001 | <0.001 | 1.30 | <0.001 | <0.001 | 0.69 | - | - |
|  | LysoPC 20:5 | 4.9 | ± | 0.3 | 9.2 | ± | 0.5 | 5.9 | ± | 0.4 | 1.61 | <0.001 | <0.001 | 0.48 | 0.030 | 0.011 | 1.48 | <0.001 | <0.001 |
|  | LysoPC 22:5 | 2.0 | ± | 0.1 | 5.0 | ± | 0.3 | 3.2 | ± | 0.2 | 1.94 | <0.001 | <0.001 | 1.12 | <0.001 | <0.001 | 1.57 | <0.001 | <0.001 |
|  | LysoPC 22:6 | 11.1 | ± | 0.3 | 33.6 | ± | 1.7 | 21.7 | ± | 1.5 | 2.20 | <0.001 | <0.001 | 1.36 | <0.001 | <0.001 | 1.85 | <0.001 | <0.001 |
|  | LysoPC 24:0 | 0.6 | ± | 0.0 | 0.6 | ± | 0.0 | 0.6 | ± | 0.0 | 0.59 | - | - | 0.68 | - | - | 1.14 | - | - |
| LysoPE | LysoPE 16:0 | 2.5 | ± | 0.1 | 1.9 | ± | 0.1 | 2.2 | ± | 0.1 | 1.41 | <0.001 | <0.001 | 0.65 | - | - | 0.71 | 0.003 | 0.002 |
|  | LysoPE 18:0 | 4.1 | ± | 0.1 | 3.0 | ± | 0.1 | 3.6 | ± | 0.1 | 1.38 | <0.001 | <0.001 | 0.58 | - | - | 0.85 | 0.001 | 0.001 |
|  | LysoPE 18:1 | 0.8 | ± | 0.0 | 0.9 | ± | 0.0 | 1.0 | ± | 0.0 | 0.95 | <0.001 | <0.001 | 1.02 | <0.001 | <0.001 | 0.88 | 0.006 | 0.003 |
|  | LysoPE 18:2 | 2.5 | ± | 0.1 | 5.1 | ± | 0.3 | 5.1 | ± | 0.4 | 1.76 | <0.001 | <0.001 | 1.42 | <0.001 | <0.001 | 0.38 | - | - |
|  | LysoPE 18:3 | 0.3 | ± | 0.0 | 0.2 | ± | 0.0 | 0.3 | ± | 0.0 | 1.16 | 0.001 | 0.001 | 0.27 | - | - | 0.76 | 0.001 | 0.001 |
|  | LysoPE 20:1 | 2.0 | ± | 0.0 | 1.9 | ± | 0.0 | 1.8 | ± | 0.0 | 0.25 |  | - | 0.95 | 0.025 | 0.009 | 1.56 | 0.022 | 0.010 |
|  | LysoPE 20:3 | 1.2 | ± | 0.0 | 1.6 | ± | 0.0 | 1.7 | ± | 0.0 | 1.74 | <0.001 | <0.001 | 1.78 | <0.001 | <0.001 | 0.95 | 0.018 | 0.009 |
|  | LysoPE 20:4 | 1.9 | ± | 0.1 | 5.9 | ± | 0.4 | 6.2 | ± | 0.4 | 2.03 | <0.001 | <0.001 | 1.77 | <0.001 | <0.001 | 0.25 | 0.032 | 0.015 |
|  | LysoPE 22:1 | 1.1 | ± | 0.0 | 1.1 | ± | 0.0 | 1.1 | ± | 0.0 | 0.52 | 0.018 | 0.012 | 0.32 | 0.004 | 0.002 | 1.72 | - | - |
|  | LysoPE 22:5 | 0.2 | ± | 0.0 | 0.4 | ± | 0.0 | 0.4 | ± | 0.0 | 1.49 | <0.001 | <0.001 | 1.39 | <0.001 | <0.001 | 0.22 | 0.046 | 0.020 |
|  | LysoPE 22:6 | 3.1 | ± | 0.1 | 10.8 | ± | 0.6 | 11.6 | ± | 0.8 | 2.22 | <0.001 | <0.001 | 1.76 | <0.001 | <0.001 | 0.24 | 0.039 | 0.017 |
| LysoPC-o | LysoPC o-16:0 | 6.3 | ± | 0.2 | 5.0 | ± | 0.1 | 5.2 | ± | 0.1 | 1.25 | <0.001 | <0.001 | 0.99 | 0.006 | 0.003 | 0.89 | - | - |
|  | LysoPC o-18:0 | 8.5 | ± | 0.2 | 6.9 | ± | 0.2 | 6.3 | ± | 0.2 | 1.19 | <0.001 | <0.001 | 1.38 | <0.001 | <0.001 | 0.97 | - | - |
| LysoPC-p | LysoPC p-18:0 | 3.5 | ± | 0.1 | 2.7 | ± | 0.1 | 3.0 | ± | 0.1 | 1.19 | <0.001 | <0.001 | 0.75 | - | - | 0.58 | 0.011 | 0.006 |
| PC | PC 14:0/18:2 | 15.7 | ± | 0.6 | 13.6 | ± | 0.5 | 12.9 | ± | 0.5 | 0.67 | - | - | 0.80 | 0.004 | 0.002 | 0.22 | - | - |
|  | PC 14:0/20:5 | 1.1 | ± | 0.1 | 1.1 | ± | 0.1 | 0.7 | ± | 0.1 | 0.14 | - | - | 0.89 | 0.001 | 0.001 | 0.87 | <0.001 | <0.001 |
|  | PC 16:0/16:0 | 50.6 | ± | 1.0 | 47.6 | ± | 1.0 | 61.1 | ± | 1.3 | 0.52 | - | - | 1.28 | <0.001 | <0.001 | 2.22 | <0.001 | <0.001 |
|  | PC 16:0/16:1 | 46.5 | ± | 2.2 | 43.9 | ± | 2.0 | 53.5 | ± | 2.1 | 0.22 | - | - | 0.51 | - | - | 1.69 | 0.046 | 0.020 |
|  | PC 16:0/18:0 | 13.8 | ± | 0.3 | 12.1 | ± | 0.3 | 13.2 | ± | 0.3 | 1.03 | 0.003 | 0.002 | 0.31 | - | - | 0.78 | - | - |
|  | PC 16:0/18:1 | 595.6 | ± | 15.2 | 582.9 | ± | 14.1 | 720.5 | ± | 16.8 | 0.15 | - | - | 1.14 | <0.001 | <0.001 | 1.97 | <0.001 | <0.001 |
|  | PC 16:0/18:2 | 1141.8 | ± | 26.0 | 1107.4 | ± | 30.7 | 1348.0 | ± | 31.8 | 0.21 | - | - | 1.05 | <0.001 | <0.001 | 1.26 | <0.001 | <0.001 |
|  | PC 16:0/18:3 | 14.5 | ± | 0.6 | 13.5 | ± | 0.5 | 14.0 | ± | 0.6 | 0.32 | - | - | 0.14 | - | - | 0.52 | - | - |
|  | PC 16:0/20:3 | 360.7 | ± | 10.0 | 338.0 | ± | 10.3 | 391.3 | ± | 10.0 | 0.39 | - | - | 0.49 | 0.003 | 0.001 | 1.06 | 0.004 | 0.002 |
|  | PC 16:0/20:4 | 469.1 | ± | 14.7 | 460.7 | ± | 15.9 | 605.5 | ± | 22.8 | 0.10 | - | - | 1.06 | <0.001 | <0.001 | 1.51 | <0.001 | <0.001 |
|  | PC 16:0/20:5 | 211.3 | ± | 11.3 | 225.9 | ± | 11.9 | 214.1 | ± | 12.6 | 0.22 | - | - | 0.04 | - | - | 0.37 | - | - |
|  | PC 16:0/22:4 | 28.6 | ± | 1.2 | 25.8 | ± | 1.0 | 28.0 | ± | 1.4 | 0.45 | - | - | 0.09 | - | - | 0.67 | - | - |
|  | PC 16:0/22:5 | 120.8 | ± | 3.1 | 119.4 | ± | 3.5 | 127.3 | ± | 4.8 | 0.08 | - | - | 0.26 | - | - | 0.51 | - | - |
|  | PC 16:0/22:6 | 600.2 | ± | 14.8 | 584.1 | ± | 16.9 | 666.8 | ± | 21.3 | 0.18 | - | - | 0.57 | 0.008 | 0.003 | 0.93 | 0.048 | 0.020 |
|  | PC 16:1/18:2 | 55.1 | ± | 2.3 | 52.0 | ± | 2.2 | 56.1 | ± | 2.4 | 0.24 | - | - | 0.07 | - | - | 0.42 | - | - |
|  | PC 16:1/20:4 | 13.0 | ± | 0.5 | 12.3 | ± | 0.5 | 11.9 | ± | 0.6 | 0.25 | - | - | 0.33 | - | - | 0.19 | - | - |
|  | PC 16:1/22:6 | 14.1 | ± | 0.4 | 13.8 | ± | 0.3 | 12.7 | ± | 0.4 | 0.14 | - | - | 0.59 | - | - | 0.90 | - | - |
|  | PC 18:0/18:1 | 133.1 | ± | 4.2 | 128.9 | ± | 4.5 | 132.4 | ± | 3.8 | 0.17 | - | - | 0.03 | - | - | 0.41 | - | - |
|  | PC 18:0/18:2 | 644.0 | ± | 16.1 | 618.7 | ± | 18.4 | 684.0 | ± | 17.6 | 0.26 | - | - | 0.38 | 0.003 | 0.001 | 0.68 | 0.013 | 0.007 |
|  | PC 18:0/20:3 | 126.7 | ± | 4.7 | 121.8 | ± | 5.0 | 130.5 | ± | 5.5 | 0.18 | - | - | 0.12 | - | - | 0.39 | - | - |
|  | PC 18:0/20:4 | 245.4 | ± | 7.6 | 256.2 | ± | 10.1 | 302.6 | ± | 10.8 | 0.21 | - | - | 0.93 | <0.001 | <0.001 | 0.74 | 0.001 | 0.001 |
|  | PC 18:0/20:5 | 111.6 | ± | 5.2 | 117.9 | ± | 5.1 | 102.7 | ± | 4.8 | 0.22 | - | - | 0.28 | - | - | 0.64 | 0.041 | 0.018 |
|  | PC 18:0/22:5 | 34.0 | ± | 1.3 | 35.4 | ± | 1.6 | 30.5 | ± | 1.4 | 0.16 | - | - | 0.42 | - | - | 0.18 | - | - |
|  | PC 18:0/22:6 | 218.5 | ± | 6.7 | 222.3 | ± | 7.4 | 225.9 | ± | 7.3 | 0.10 | - | - | 0.17 | - | - | 0.71 | 0.020 | 0.010 |
|  | PC 18:1/18:2 | 197.1 | ± | 5.8 | 188.7 | ± | 5.7 | 220.7 | ± | 6.0 | 0.26 | - | - | 0.63 | <0.001 | <0.001 | 0.20 | - | - |
|  | PC 18:1/22:6 | 27.6 | ± | 0.8 | 27.0 | ± | 0.9 | 26.8 | ± | 0.8 | 0.14 | - | - | 0.16 | - | - | 0.91 | <0.001 | <0.001 |
|  | PC 18:2/20:4 | 36.5 | ± | 1.2 | 35.4 | ± | 1.1 | 32.3 | ± | 1.3 | 0.17 | - | - | 0.53 | - | - | 0.54 | - | - |
|  | PC 20:0/18:2 | 20.0 | ± | 0.5 | 19.6 | ± | 0.6 | 19.6 | ± | 0.5 | 0.14 | - | - | 0.12 | - | - | 0.60 | - | - |
|  | PC 20:4/20:4 | 6.2 | ± | 0.2 | 5.7 | ± | 0.2 | 5.4 | ± | 0.2 | 0.56 | - | - | 0.70 | 0.047 | 0.017 | 0.47 | - | - |
|  | PC 20:4/22:6 | 1.9 | ± | 0.1 | 1.8 | ± | 0.0 | 1.8 | ± | 0.1 | 0.49 | - | - | 0.44 | - | - | 0.48 | - | - |
| PE | PE 16:0/20:4 | 5.8 | ± | 0.2 | 4.1 | ± | 0.2 | 7.1 | ± | 0.3 | 1.19 | <0.001 | <0.001 | 1.05 | 0.001 | 0.001 | 1.39 | <0.001 | <0.001 |
|  | PE 18:0/18:1 | 1.8 | ± | 0.1 | 2.1 | ± | 0.1 | 1.8 | ± | 0.1 | 0.67 | 0.011 | - | 0.19 | - | - | 0.55 | 0.004 | 0.002 |
|  | PE 18:0/20:3 | 0.9 | ± | 0.0 | 0.7 | ± | 0.0 | 0.8 | ± | 0.0 | 0.95 | <0.001 | <0.001 | 0.81 | - | - | 0.38 | - | - |
|  | PE 18:0/20:4 | 9.2 | ± | 0.4 | 6.5 | ± | 0.3 | 10.3 | ± | 0.5 | 1.14 | <0.001 | <0.001 | 0.63 | 0.019 | 0.007 | 1.26 | <0.001 | <0.001 |
|  | PE 18:0/20:5 | 1.9 | ± | 0.1 | 1.5 | ± | 0.1 | 1.5 | ± | 0.1 | 0.59 | 0.002 | 0.002 | 0.86 | - | - | 0.03 | - | - |
|  | PE 18:1/18:2 | 1.0 | ± | 0.0 | 0.7 | ± | 0.0 | 1.0 | ± | 0.1 | 0.86 | 0.001 | 0.001 | 0.18 | - | - | 0.82 | 0.001 | 0.001 |
| PI | PI 16:0/20:4 | 5.1 | ± | 0.2 | 5.7 | ± | 0.2 | 4.1 | ± | 0.2 | 0.46 | 0.034 | - | 0.94 | <0.001 | <0.001 | 0.95 | <0.001 | <0.001 |
|  | PI 18:0/18:1 | 3.1 | ± | 0.1 | 3.5 | ± | 0.1 | 2.3 | ± | 0.1 | 0.48 | 0.014 | - | 1.54 | <0.001 | <0.001 | 1.24 | <0.001 | <0.001 |
|  | PI 18:0/18:2 | 20.4 | ± | 0.9 | 23.8 | ± | 1.3 | 17.8 | ± | 0.8 | 0.49 | 0.032 | - | 0.64 | - | - | 0.82 | <0.001 | <0.001 |
|  | PI 18:0/20:3 | 6.0 | ± | 0.2 | 7.7 | ± | 0.4 | 5.2 | ± | 0.3 | 0.86 | 0.022 | - | 0.73 | 0.022 | 0.008 | 1.07 | <0.001 | <0.001 |
|  | PI 18:0/20:4 | 49.6 | ± | 1.8 | 58.9 | ± | 2.1 | 43.0 | ± | 1.6 | 0.75 | 0.029 | - | 0.80 | 0.001 | 0.001 | 1.14 | <0.001 | <0.001 |
|  | PI 18:0/22:5 | 2.1 | ± | 0.1 | 2.7 | ± | 0.1 | 1.8 | ± | 0.1 | 0.86 | 0.002 | 0.002 | 0.72 | 0.008 | 0.003 | 1.19 | <0.001 | <0.001 |
|  | PI 18:0/22:6 | 8.0 | ± | 0.5 | 9.7 | ± | 0.6 | 7.0 | ± | 0.5 | 0.48 | 0.006 | - | 0.41 | - | - | 0.69 | <0.001 | <0.001 |
|  | PI 18:1/18:2 | 2.0 | ± | 0.1 | 2.3 | ± | 0.1 | 1.5 | ± | 0.1 | 0.49 | 0.023 | - | 1.53 | <0.001 | <0.001 | 1.20 | <0.001 | <0.001 |
|  | PI 18:1/20:4 | 1.9 | ± | 0.1 | 2.2 | ± | 0.1 | 1.5 | ± | 0.1 | 0.56 | 0.005 | 0.004 | 1.35 | <0.001 | <0.001 | 1.19 | <0.001 | <0.001 |
| PC-o | PC o-16:0/18:2 | 17.8 | ± | 0.6 | 16.3 | ± | 0.7 | 19.0 | ± | 0.6 | 0.41 | - | - | 0.29 | - | - | 1.06 | - | - |
|  | PC o-16:0/20:4 | 57.4 | ± | 1.9 | 56.5 | ± | 2.0 | 61.8 | ± | 1.9 | 0.09 | - | - | 0.37 | - | - | 0.59 | 0.041 | 0.018 |
|  | PC o-16:0/22:6 | 23.3 | ± | 0.7 | 23.0 | ± | 0.7 | 24.3 | ± | 0.7 | 0.08 | - | - | 0.21 | - | - | 0.59 | - | - |
|  | PC o-18:0/16:0 | 0.9 | ± | 0.0 | 0.9 | ± | 0.0 | 1.1 | ± | 0.0 | 0.35 | - | - | 0.72 | <0.001 | <0.001 | 1.10 | <0.001 | <0.001 |
|  | PC o-18:0/18:2 | 36.8 | ± | 1.0 | 36.9 | ± | 1.2 | 38.2 | ± | 1.1 | 0.02 | - | - | 0.22 | - | - | 0.46 | - | - |
|  | PC o-18:0/20:4 | 13.6 | ± | 0.5 | 13.6 | ± | 0.6 | 15.5 | ± | 0.5 | 0.00 | - | - | 0.64 | 0.001 | 0.001 | 0.63 | 0.010 | 0.005 |
|  | PC o-18:0/22:6 | 6.7 | ± | 0.2 | 6.9 | ± | 0.2 | 7.2 | ± | 0.2 | 0.18 | - | - | 0.33 | - | - | 0.73 | - | - |
|  | PC o-20:0/20:4 | 2.7 | ± | 0.1 | 2.7 | ± | 0.1 | 3.3 | ± | 0.1 | 0.10 | - | - | 0.97 | <0.001 | <0.001 | 0.91 | <0.001 | <0.001 |
| PC-p | PC p-16:0/16:0 | 6.8 | ± | 0.2 | 6.1 | ± | 0.2 | 7.5 | ± | 0.2 | 0.55 | - | - | 0.52 | 0.002 | 0.001 | 1.20 | <0.001 | <0.001 |
|  | PC p-16:0/20:4 | 37.8 | ± | 1.1 | 34.0 | ± | 1.0 | 41.0 | ± | 1.2 | 0.61 | - | - | 0.46 | 0.012 | 0.005 | 1.07 | <0.001 | <0.001 |
|  | PC p-18:0/16:0 | 18.3 | ± | 0.5 | 17.6 | ± | 0.4 | 21.7 | ± | 0.6 | 0.26 | - | - | 0.93 | <0.001 | <0.001 | 1.38 | <0.001 | <0.001 |
|  | PC p-18:0/18:1 | 49.0 | ± | 1.4 | 49.2 | ± | 1.4 | 56.1 | ± | 1.5 | 0.03 | - | - | 0.75 | 0.001 | 0.001 | 0.77 | 0.001 | 0.001 |
|  | PC p-18:0/18:2 | 12.9 | ± | 0.5 | 12.4 | ± | 0.4 | 13.1 | ± | 0.4 | 0.19 | - | - | 0.09 | - | - | 0.69 | - | - |
| PE-p | PE p-16:0/20:3 | 3.9 | ± | 0.2 | 4.6 | ± | 0.3 | 2.6 | ± | 0.2 | 0.48 | 0.038 | - | 1.45 | <0.001 | <0.001 | 1.22 | <0.001 | <0.001 |
|  | PE p-16:0/20:4 | 8.0 | ± | 0.5 | 9.1 | ± | 0.5 | 5.5 | ± | 0.3 | 0.39 | - | - | 1.35 | <0.001 | <0.001 | 1.15 | <0.001 | <0.001 |
|  | PE p-16:0/20:5 | 4.1 | ± | 0.4 | 5.2 | ± | 0.4 | 2.4 | ± | 0.3 | 0.43 | - | - | 1.19 | 0.001 | 0.001 | 1.03 | <0.001 | <0.001 |
|  | PE p-16:0/22:6 | 10.1 | ± | 0.6 | 12.7 | ± | 0.8 | 8.3 | ± | 0.5 | 0.62 | 0.029 | - | 0.73 | 0.018 | 0.007 | 0.96 | <0.001 | <0.001 |
|  | PE p-18:0/18:2 | 3.3 | ± | 0.2 | 4.0 | ± | 0.2 | 2.2 | ± | 0.2 | 0.52 | 0.023 | - | 1.29 | 0.001 | 0.001 | 1.14 | <0.001 | <0.001 |
|  | PE p-18:0/20:4 | 11.7 | ± | 0.8 | 14.5 | ± | 1.0 | 8.3 | ± | 0.5 | 0.50 | 0.018 | - | 1.16 | <0.001 | <0.001 | 1.09 | <0.001 | <0.001 |
|  | PE p-18:0/22:6 | 7.6 | ± | 0.5 | 9.7 | ± | 0.6 | 5.7 | ± | 0.4 | 0.60 | 0.016 | - | 0.95 | 0.001 | 0.001 | 1.13 | <0.001 | <0.001 |
|  | PE p-18:1/20:4 | 10.5 | ± | 0.6 | 12.5 | ± | 0.7 | 7.2 | ± | 0.4 | 0.50 | - | - | 1.38 | <0.001 | <0.001 | 1.19 | <0.001 | <0.001 |
|  | PE p-18:1/22:6 | 4.5 | ± | 0.3 | 5.6 | ± | 0.4 | 3.3 | ± | 0.2 | 0.52 | - | - | 1.09 | 0.002 | 0.001 | 1.05 | <0.001 | <0.001 |
| DG | DG 16:0/18:1 | 8.5 | ± | 0.5 | 5.2 | ± | 0.3 | 8.0 | ± | 0.4 | 1.33 | <0.001 | <0.001 | 0.19 | 0.690 | - | 1.70 | <0.001 | <0.001 |
|  | DG 18:1/18:1 | 376.3 | ± | 7.0 | 406.2 | ± | 8.9 | 439.8 | ± | 8.3 | 0.65 | 0.003 | 0.002 | 1.20 | <0.001 | <0.001 | 1.21 | 0.001 | 0.001 |
|  | DG 18:1/18:2 | 113.4 | ± | 2.1 | 121.6 | ± | 2.8 | 131.6 | ± | 2.6 | 0.58 | 0.007 | - | 1.13 | <0.001 | <0.001 | 1.21 | <0.001 | <0.001 |
| SM | SM d16:1/23:0 | 14.3 | ± | 0.4 | 14.3 | ± | 0.4 | 15.1 | ± | 0.5 | 0.01 | - | - | 0.27 | - | - | 0.33 | - | - |
|  | SM d18:1/14:0 | 35.0 | ± | 0.9 | 33.1 | ± | 0.9 | 37.6 | ± | 1.1 | 0.39 | - | - | 0.40 | - | - | 0.79 | - | - |
|  | SM d18:1/16:0 | 301.7 | ± | 6.3 | 296.0 | ± | 7.1 | 355.7 | ± | 8.2 | 0.15 | - | - | 1.09 | <0.001 | <0.001 | 1.24 | <0.001 | <0.001 |
|  | SM d18:1/18:0 | 51.1 | ± | 1.3 | 52.6 | ± | 1.4 | 67.3 | ± | 2.4 | 0.20 | - | - | 1.20 | <0.001 | <0.001 | 1.26 | <0.001 | <0.001 |
|  | SM d18:1/20:0 | 37.9 | ± | 1.0 | 39.2 | ± | 1.0 | 43.5 | ± | 1.5 | 0.24 | - | - | 0.71 | 0.006 | 0.003 | 0.60 | - | - |
|  | SM d18:1/22:0 | 87.1 | ± | 2.4 | 86.7 | ± | 2.8 | 98.5 | ± | 3.6 | 0.03 | - | - | 0.59 | 0.007 | 0.003 | 0.62 | - | - |
|  | SM d18:1/23:0 | 39.2 | ± | 1.0 | 38.1 | ± | 1.1 | 43.1 | ± | 1.5 | 0.18 | - | - | 0.49 | - | - | 0.67 | - | - |
|  | SM d18:1/24:0 | 69.2 | ± | 2.1 | 66.8 | ± | 1.9 | 76.2 | ± | 2.8 | 0.21 | - | - | 0.46 | 0.017 | 0.007 | 0.65 | - | - |
|  | SM d18:1/24:1 | 43.5 | ± | 1.0 | 43.5 | ± | 1.0 | 52.1 | ± | 1.2 | 0.00 | - | - | 1.11 | <0.001 | <0.001 | 1.22 | <0.001 | <0.001 |
|  | SM d18:1/24:2 | 17.8 | ± | 0.5 | 18.0 | ± | 0.5 | 21.9 | ± | 0.6 | 0.08 | - | - | 1.06 | <0.001 | <0.001 | 1.07 | <0.001 | <0.001 |
|  | SM d18:2/16:0 | 57.7 | ± | 1.4 | 55.4 | ± | 1.5 | 67.4 | ± | 1.7 | 0.27 | - | - | 0.94 | <0.001 | <0.001 | 1.24 | <0.001 | <0.001 |
|  | SM d18:2/18:0 | 30.5 | ± | 0.9 | 31.0 | ± | 0.9 | 38.3 | ± | 1.3 | 0.10 | - | - | 1.04 | <0.001 | <0.001 | 1.14 | 0.001 | 0.001 |
|  | SM d18:2/20:0 | 17.5 | ± | 0.5 | 18.1 | ± | 0.4 | 20.1 | ± | 0.5 | 0.23 | 0.029 | - | 0.82 | 0.001 | 0.001 | 0.81 | 0.021 | 0.010 |
|  | SM d18:2/22:0 | 58.5 | ± | 1.5 | 59.1 | ± | 1.5 | 64.7 | ± | 1.6 | 0.08 | - | - | 0.64 | 0.003 | 0.001 | 0.69 | - | - |
|  | SM d18:2/23:0 | 29.6 | ± | 0.8 | 29.9 | ± | 0.7 | 32.8 | ± | 0.8 | 0.08 | - | - | 0.65 | 0.017 | 0.007 | 0.65 | - | - |
|  | SM d18:2/24:0 | 196.0 | ± | 5.0 | 196.4 | ± | 4.9 | 239.5 | ± | 6.3 | 0.02 | - | - | 1.12 | <0.001 | <0.001 | 1.26 | <0.001 | <0.001 |
|  | SM d18:2/24:1 | 80.8 | ± | 2.3 | 81.8 | ± | 2.2 | 97.9 | ± | 2.6 | 0.08 | - | - | 1.04 | <0.001 | <0.001 | 1.07 | <0.001 | <0.001 |
| Cer | Cer d18:0/24:1 | 21.3 | ± | 0.4 | 22.2 | ± | 0.5 | 24.2 | ± | 0.5 | 0.38 | 0.025 | - | 0.97 | <0.001 | <0.001 | 1.01 | 0.002 | 0.001 |
|  | Cer d18:1/22:1 | 1.4 | ± | 0.0 | 1.5 | ± | 0.0 | 1.6 | ± | 0.0 | 0.62 | 0.002 | 0.002 | 1.20 | <0.001 | <0.001 | 1.06 | <0.001 | <0.001 |
|  | Cer d18:1/24:1 | 7.0 | ± | 0.1 | 7.1 | ± | 0.2 | 8.3 | ± | 0.2 | 0.17 | - | - | 1.19 | <0.001 | <0.001 | 1.04 | <0.001 | <0.001 |
| Glucer | Glucer d18:1/16:0 | 0.8 | ± | 0.0 | 0.8 | ± | 0.0 | 1.0 | ± | 0.0 | 0.37 | - | - | 1.01 | <0.001 | <0.001 | 1.47 | <0.001 | <0.001 |
|  | Glucer d18:1/22:0 | 1.5 | ± | 0.1 | 1.3 | ± | 0.0 | 1.5 | ± | 0.1 | 0.62 | - | - | 0.16 | - | - | 0.85 | 0.014 | 0.007 |
| CE | CE 18:2 | 8.9 | ± | 0.4 | 8.4 | ± | 0.3 | 9.2 | ± | 0.3 | 0.25 | - | - | 0.18 | - | - | 0.83 | 0.012 | 0.006 |
|  | CE 18:3 | 2.2 | ± | 0.1 | 2.3 | ± | 0.1 | 2.3 | ± | 0.1 | 0.38 | 0.032 | - | 0.34 | 0.010 | 0.004 | 1.04 | - | - |
|  | CE 20:3 | 0.7 | ± | 0.0 | 0.7 | ± | 0.0 | 0.7 | ± | 0.0 | 0.30 | - | - | 0.54 | 0.038 | 0.014 | 0.50 | - | - |
|  | CE 20:4 | 5.6 | ± | 0.3 | 6.7 | ± | 0.3 | 7.0 | ± | 0.3 | 0.67 | 0.004 | 0.003 | 0.79 | 0.004 | 0.002 | 0.66 | - | - |
|  | CE 20:5 | 20.6 | ± | 0.8 | 21.8 | ± | 0.8 | 22.9 | ± | 0.7 | 0.27 | - | - | 0.47 | 0.009 | 0.004 | 0.96 | - | - |
|  | CE 22:6 | 4.2 | ± | 0.2 | 5.9 | ± | 0.2 | 5.2 | ± | 0.2 | 1.29 | <0.001 | <0.001 | 0.82 | 0.001 | 0.001 | 1.53 | - | - |

The data are presented as the mean ± SE. Each p value was obtained from general linear model after adjustment for age, sex, BMI, LDL cholesterol, and fasting glucose. False discovery rate (FDR) q-value was calculated to correct for multiple comparisons.
