# Supplementary material for: Alteration in Metabolic Signature and Lipid Metabolism in Patients with Angina Pectoris and Myocardial Infarction
Source: PLoS One. 2015 Aug 10;10(8):e0135228. doi: 10.1371/journal.pone.0135228 (PMC4530944; doi:10.1371/journal.pone.0135228)
Supplement: S6 Table — (DOCX) [file pone.0135228.s007.docx]

**S6 Table. Levels of individual lipid species in patients with CAD. Statistical significance was presented in bold (p <0.05)** ^a^**.**

|  | **Stable angina** | | | **Unstable angina** | | | **MI** | | | **P1^b^** | **P2 ^c^** | **P3 ^d^** |
| --- | --- | --- | --- | --- | --- | --- | --- | --- | --- | --- | --- | --- |
| FFA 16:0 | 485.15 | **±** | 23.59 | 421.47 | **±** | 40.25 | 279.31 | **±** | 12.24 | 0.612 | **<0.001** | **<0.001** |
| FFA 16:1 | 134.12 | **±** | 7.44 | 124.85 | **±** | 14.76 | 72.21 | **±** | 4.20 | 0.969 | **<0.001** | **<0.001** |
| FFA 18:0 | 221.71 | **±** | 10.40 | 190.85 | **±** | 15.03 | 133.60 | **±** | 5.32 | 0.801 | **<0.001** | **<0.001** |
| FFA 18:1 | 679.27 | **±** | 35.17 | 609.82 | **±** | 53.11 | 377.60 | **±** | 17.67 | 0.902 | **<0.001** | **<0.001** |
| FFA 18:2 | 428.42 | **±** | 25.03 | 416.65 | **±** | 45.58 | 229.47 | **±** | 12.60 | 0.695 | **<0.001** | **<0.001** |
| FFA 18:3 | 93.32 | **±** | 6.95 | 96.51 | **±** | 12.55 | 37.60 | **±** | 3.31 | 0.461 | **<0.001** | **<0.001** |
| FFA 20:1 | 17.37 | **±** | 1.53 | 14.98 | **±** | 1.80 | 9.87 | **±** | 0.84 | 0.822 | **<0.001** | **0.014** |
| FFA 20:2 | 14.92 | **±** | 1.03 | 14.01 | **±** | 1.71 | 6.95 | **±** | 0.44 | 0.751 | **<0.001** | **<0.001** |
| FFA 20:3 | 19.17 | **±** | 1.31 | 17.37 | **±** | 2.27 | 7.97 | **±** | 0.61 | 0.892 | **<0.001** | **<0.001** |
| FFA 22:3 | 0.38 | **±** | 0.02 | 0.34 | **±** | 0.04 | 0.20 | **±** | 0.01 | 0.720 | **<0.001** | **<0.001** |
| FFA 22:6 | 113.18 | **±** | 8.79 | 98.21 | **±** | 16.40 | 44.02 | **±** | 4.31 | 0.931 | **<0.001** | **<0.001** |
| FFA 24:5 | 3.39 | **±** | 0.35 | 2.74 | **±** | 0.57 | 1.22 | **±** | 0.16 | 0.248 | **<0.001** | **0.012** |
| FFA 24:6 | 2.44 | **±** | 0.23 | 1.39 | **±** | 0.22 | 0.64 | **±** | 0.09 | 0.132 | **<0.001** | **0.014** |
| LysoPC 16:0 | 294.87 | **±** | 6.54 | 303.21 | **±** | 14.37 | 321.65 | **±** | 10.00 | 0.808 | 0.186 | 0.137 |
| LysoPC 18:0 | 120.06 | **±** | 3.20 | 130.30 | **±** | 7.29 | 120.86 | **±** | 4.54 | 0.497 | 0.874 | 0.999 |
| LysoPC 20:3 | 14.90 | **±** | 0.81 | 16.11 | **±** | 1.76 | 11.73 | **±** | 0.65 | 0.670 | **0.043** | 0.243 |
| LysoPC 20:4 | 31.03 | **±** | 1.67 | 34.55 | **±** | 4.40 | 28.98 | **±** | 1.51 | 0.709 | **<0.001** | 0.884 |
| LysoPC 22:5 | 5.04 | **±** | 0.33 | 4.75 | **±** | 0.49 | 3.22 | **±** | 0.21 | 0.275 | **<0.001** | 0.064 |
| LysoPC 22:6 | 33.39 | **±** | 1.84 | 34.60 | **±** | 4.04 | 21.66 | **±** | 1.49 | 0.714 | **<0.001** | 0.067 |
| LysoPE 18:2 | 4.64 | **±** | 0.26 | 6.79 | **±** | 0.81 | 5.14 | **±** | 0.35 | 0.236 | **0.045** | 0.564 |
| LysoPE 20:3 | 1.58 | **±** | 0.04 | 1.76 | **±** | 0.14 | 1.67 | **±** | 0.04 | 0.384 | **0.009** | 0.365 |
| LysoPE 20:4 | 5.60 | **±** | 0.26 | 7.16 | **±** | 1.33 | 6.21 | **±** | 0.41 | 0.566 | **0.019** | 0.454 |
| LysoPE 22:5 | 0.38 | **±** | 0.02 | 0.47 | **±** | 0.09 | 0.43 | **±** | 0.03 | 0.373 | 0.075 | 0.173 |
| LysoPE 22:6 | 10.33 | **±** | 0.45 | 12.42 | **±** | 2.07 | 11.65 | **±** | 0.83 | 0.922 | 0.051 | 0.168 |
| LysoPC o-18:0 | 6.87 | **±** | 0.21 | 7.20 | **±** | 0.47 | 6.27 | **±** | 0.21 | 0.076 | 0.095 | 0.100 |

The data are presented as the mean ± SE.

^a^ Significance determined by general linear model (GLM) after adjustments for age, sex, BMI, LDL cholesterol, and fasting glucose.

^b^ P1 : Compared with between stable angina and unstable angina patients.

^c^ P2 : Compared with between stable angina and MI patients.

^d^ P3 : Compared with between unstable angina and MI patients.
