# Supplementary material for: Alteration in Metabolic Signature and Lipid Metabolism in Patients with Angina Pectoris and Myocardial Infarction
Source: PLoS One. 2015 Aug 10;10(8):e0135228. doi: 10.1371/journal.pone.0135228 (PMC4530944; doi:10.1371/journal.pone.0135228)
Supplement: S7 Table — (DOCX) [file pone.0135228.s008.docx]

**S7 Table. Odds ratio for angina or MI of individual lipid species**

| Class | Metabolites | Angina | | | | | | | |  | MI | | | | | | | |
| --- | --- | --- | --- | --- | --- | --- | --- | --- | --- | --- | --- | --- | --- | --- | --- | --- | --- | --- |
|  |  | OR per SE | (95%CI) | | | | | p value | q value |  | OR per SE | (95%CI) | | | | | p value | q value |
| FFA | FFA 16:0 | 1.83 | ( | 1.40 | - | 2.40 | ) | <0.001 | <0.001 |  | 1.23 | ( | 1.08 | - | 1.41 | ) | 0.002 | 0.001 |
|  | FFA 16:1 | 1.95 | ( | 1.41 | - | 2.69 | ) | <0.001 | <0.001 |  | 1.45 | ( | 1.20 | - | 1.74 | ) | <0.001 | <0.001 |
|  | FFA 18:0 | 1.41 | ( | 1.23 | - | 1.62 | ) | <0.001 | <0.001 |  | 1.09 | ( | 0.99 | - | 1.19 | ) | - | - |
|  | FFA 18:1 | 1.86 | ( | 1.39 | - | 2.48 | ) | <0.001 | <0.001 |  | 1.30 | ( | 1.12 | - | 1.51 | ) | 0.001 | 0.001 |
|  | FFA 18:2 | 1.68 | ( | 1.34 | - | 2.12 | ) | <0.001 | <0.001 |  | 1.27 | ( | 1.11 | - | 1.46 | ) | 0.001 | 0.001 |
|  | FFA 18:3 | 1.77 | ( | 1.37 | - | 2.28 | ) | <0.001 | <0.001 |  | 1.29 | ( | 1.07 | - | 1.56 | ) | 0.008 | 0.004 |
|  | FFA 20:1 | 1.71 | ( | 1.37 | - | 2.14 | ) | <0.001 | <0.001 |  | 1.31 | ( | 1.12 | - | 1.55 | ) | 0.001 | 0.001 |
|  | FFA 20:2 | 1.99 | ( | 1.44 | - | 2.76 | ) | <0.001 | <0.001 |  | 1.42 | ( | 1.16 | - | 1.74 | ) | 0.001 | 0.001 |
|  | FFA 20:3 | 2.05 | ( | 1.47 | - | 2.86 | ) | <0.001 | <0.001 |  | 1.28 | ( | 1.03 | - | 1.58 | ) | 0.025 | 0.011 |
|  | FFA 20:4 | 1.34 | ( | 1.18 | - | 1.52 | ) | <0.001 | <0.001 |  | 1.06 | ( | 0.95 | - | 1.18 | ) | - | - |
|  | FFA 20:5 | 1.58 | ( | 1.31 | - | 1.92 | ) | <0.001 | <0.001 |  | 1.08 | ( | 0.96 | - | 1.22 | ) | - | - |
|  | FFA 22:3 | 1.65 | ( | 1.32 | - | 2.06 | ) | <0.001 | <0.001 |  | 1.24 | ( | 1.05 | - | 1.45 | ) | 0.009 | 0.005 |
|  | FFA 22:6 | 1.60 | ( | 1.31 | - | 1.95 | ) | <0.001 | <0.001 |  | 1.13 | ( | 0.99 | - | 1.28 | ) | - | - |
|  | FFA 24:5 | 3.84 | ( | 1.89 | - | 7.78 | ) | <0.001 | <0.001 |  | 1.90 | ( | 1.23 | - | 2.95 | ) | 0.004 | 0.002 |
|  | FFA 24:6 | 3.48 | ( | 1.85 | - | 6.56 | ) | <0.001 | <0.001 |  | 1.44 | ( | 1.00 | - | 2.07 | ) | 0.048 | 0.020 |
| LysoPC | LysoPC 14:0 | 0.84 | ( | 0.78 | - | 0.90 | ) | <0.001 | <0.001 |  | 0.82 | ( | 0.75 | - | 0.90 | ) | <0.001 | <0.001 |
|  | LysoPC 16:0 | 0.77 | ( | 0.70 | - | 0.85 | ) | <0.001 | <0.001 |  | 0.91 | ( | 0.86 | - | 0.97 | ) | 0.002 | 0.001 |
|  | LysoPC 16:1 | 0.96 | ( | 0.92 | - | 1.00 | ) | - | - |  | 0.96 | ( | 0.91 | - | 1.01 | ) | - | - |
|  | LysoPC 18:0 | 0.81 | ( | 0.74 | - | 0.88 | ) | <0.001 | <0.001 |  | 0.88 | ( | 0.82 | - | 0.94 | ) | <0.001 | <0.001 |
|  | LysoPC 18:1 | 1.04 | ( | 0.99 | - | 1.08 | ) | - | - |  | 1.03 | ( | 0.98 | - | 1.08 | ) |  |  |
|  | LysoPC 18:2 | 1.30 | ( | 1.17 | - | 1.43 | ) | <0.001 | <0.001 |  | 1.13 | ( | 1.05 | - | 1.22 | ) | 0.001 | 0.001 |
|  | LysoPC 18:3 | 1.11 | ( | 1.05 | - | 1.18 | ) | <0.001 | <0.001 |  | 1.00 | ( | 0.95 | - | 1.06 | ) | - | - |
|  | LysoPC 18:4 | 0.99 | ( | 0.95 | - | 1.03 | ) | - | - |  | 0.97 | ( | 0.92 | - | 1.03 | ) | - | - |
|  | LysoPC 20:1 | 0.99 | ( | 0.95 | - | 1.03 | ) | - | - |  | 1.02 | ( | 0.97 | - | 1.07 | ) | - | - |
|  | LysoPC 20:3 | 1.53 | ( | 1.29 | - | 1.81 | ) | <0.001 | <0.001 |  | 1.39 | ( | 1.17 | - | 1.64 | ) | <0.001 | <0.001 |
|  | LysoPC 20:4 | 1.62 | ( | 1.34 | - | 1.96 | ) | <0.001 | <0.001 |  | 1.37 | ( | 1.17 | - | 1.61 | ) | <0.001 | <0.001 |
|  | LysoPC 20:5 | 1.20 | ( | 1.11 | - | 1.31 | ) | <0.001 | <0.001 |  | 1.07 | ( | 1.00 | - | 1.15 | ) | - | - |
|  | LysoPC 22:5 | 1.76 | ( | 1.40 | - | 2.20 | ) | <0.001 | <0.001 |  | 1.35 | ( | 1.15 | - | 1.59 | ) | <0.001 | <0.001 |
|  | LysoPC 22:6 | 1.78 | ( | 1.38 | - | 2.30 | ) | <0.001 | <0.001 |  | 1.45 | ( | 1.20 | - | 1.74 | ) | <0.001 | <0.001 |
|  | LysoPC 24:0 | 0.98 | ( | 0.94 | - | 1.03 | ) | - | - |  | 0.98 | ( | 0.93 | - | 1.03 | ) | - | - |
| LysoPE | LysoPE 16:0 | 0.87 | ( | 0.81 | - | 0.93 | ) | <0.001 | <0.001 |  | 1.00 | ( | 0.94 | - | 1.06 | ) | - | - |
|  | LysoPE 18:0 | 0.86 | ( | 0.80 | - | 0.92 | ) | <0.001 | <0.001 |  | 1.01 | ( | 0.95 | - | 1.07 | ) | - | - |
|  | LysoPE 18:1 | 1.10 | ( | 1.04 | - | 1.17 | ) | 0.001 | 0.001 |  | 1.14 | ( | 1.06 | - | 1.22 | ) | <0.001 | <0.001 |
|  | LysoPE 18:2 | 1.41 | ( | 1.23 | - | 1.61 | ) | <0.001 | <0.001 |  | 1.30 | ( | 1.14 | - | 1.49 | ) | <0.001 | <0.001 |
|  | LysoPE 18:3 | 0.92 | ( | 0.87 | - | 0.97 | ) | 0.001 | 0.001 |  | 1.01 | ( | 0.96 | - | 1.07 | ) | - | - |
|  | LysoPE 20:1 | 0.98 | ( | 0.94 | - | 1.03 | ) | - | - |  | 0.94 | ( | 0.88 | - | 0.99 | ) | 0.025 | 0.011 |
|  | LysoPE 20:3 | 1.44 | ( | 1.25 | - | 1.67 | ) | <0.001 | <0.001 |  | 1.74 | ( | 1.33 | - | 2.27 | ) | <0.001 | <0.001 |
|  | LysoPE 20:4 | 2.74 | ( | 1.69 | - | 4.43 | ) | <0.001 | <0.001 |  | 1.79 | ( | 1.32 | - | 2.44 | ) | <0.001 | <0.001 |
|  | LysoPE 22:1 | 1.05 | ( | 1.00 | - | 1.09 | ) | 0.033 | 0.019 |  | 1.06 | ( | 1.01 | - | 1.12 | ) | 0.025 | 0.011 |
|  | LysoPE 22:5 | 1.55 | ( | 1.30 | - | 1.84 | ) | <0.001 | <0.001 |  | 1.43 | ( | 1.20 | - | 1.71 | ) | <0.001 | <0.001 |
|  | LysoPE 22:6 | 2.52 | ( | 1.61 | - | 3.93 | ) | <0.001 | <0.001 |  | 1.65 | ( | 1.25 | - | 2.18 | ) | <0.001 | <0.001 |
| LysoPC-o | LysoPC o-16:0 | 0.90 | ( | 0.85 | - | 0.95 | ) | <0.001 | <0.001 |  | 0.93 | ( | 0.88 | - | 0.99 | ) | 0.015 | 0.007 |
|  | LysoPC o-18:0 | 0.89 | ( | 0.84 | - | 0.94 | ) | <0.001 | <0.001 |  | 0.86 | ( | 0.81 | - | 0.93 | ) | <0.001 | <0.001 |
| LysoPC-p | LysoPC p-18:0 | 0.88 | ( | 0.82 | - | 0.94 | ) | <0.001 | <0.001 |  | 0.96 | ( | 0.91 | - | 1.01 | ) | - | - |
| PC | PC 14:0/18:2 | 0.95 | ( | 0.90 | - | 0.99 | ) | 0.027 | 0.016 |  | 0.92 | ( | 0.87 | - | 0.98 | ) | 0.009 | 0.005 |
|  | PC 14:0/20:5 | 0.98 | ( | 0.94 | - | 1.02 | ) | - | - |  | 0.93 | ( | 0.88 | - | 0.98 | ) | 0.007 | 0.004 |
|  | PC 16:0/16:0 | 0.98 | ( | 0.92 | - | 1.04 | ) | - | - |  | 1.15 | ( | 1.07 | - | 1.23 | ) | <0.001 | <0.001 |
|  | PC 16:0/16:1 | 0.99 | ( | 0.95 | - | 1.03 | ) | - | - |  | 1.03 | ( | 0.98 | - | 1.08 | ) | - | - |
|  | PC 16:0/18:0 | 0.92 | ( | 0.86 | - | 0.97 | ) | 0.005 | 0.003 |  | 0.99 | ( | 0.94 | - | 1.05 | ) | - | - |
|  | PC 16:0/18:1 | 1.01 | ( | 0.96 | - | 1.06 | ) | - | - |  | 1.17 | ( | 1.08 | - | 1.27 | ) | <0.001 | <0.001 |
|  | PC 16:0/18:2 | 1.00 | ( | 0.95 | - | 1.05 | ) | - | - |  | 1.15 | ( | 1.07 | - | 1.24 | ) | <0.001 | <0.001 |
|  | PC 16:0/18:3 | 1.00 | ( | 0.96 | - | 1.05 | ) | - | - |  | 1.00 | ( | 0.95 | - | 1.04 | ) | - | - |
|  | PC 16:0/20:3 | 0.98 | ( | 0.94 | - | 1.03 | ) | - | - |  | 1.07 | ( | 1.01 | - | 1.14 | ) | 0.017 | 0.008 |
|  | PC 16:0/20:4 | 1.00 | ( | 0.94 | - | 1.06 | ) | - | - |  | 1.16 | ( | 1.06 | - | 1.26 | ) | 0.001 | 0.001 |
|  | PC 16:0/20:5 | 1.00 | ( | 0.96 | - | 1.05 | ) | - | - |  | 1.00 | ( | 0.95 | - | 1.05 | ) | - | - |
|  | PC 16:0/22:4 | 0.98 | ( | 0.94 | - | 1.03 | ) | - | - |  | 0.99 | ( | 0.94 | - | 1.05 | ) | - | - |
|  | PC 16:0/22:5 | 1.01 | ( | 0.96 | - | 1.06 | ) | - | - |  | 1.04 | ( | 0.98 | - | 1.09 | ) | - | - |
|  | PC 16:0/22:6 | 0.99 | ( | 0.94 | - | 1.04 | ) | - | - |  | 1.05 | ( | 1.00 | - | 1.11 | ) | - | - |
|  | PC 16:1/18:2 | 0.99 | ( | 0.95 | - | 1.03 | ) | - | - |  | 1.00 | ( | 0.96 | - | 1.05 | ) | - | - |
|  | PC 16:1/20:4 | 0.99 | ( | 0.94 | - | 1.03 | ) | - | - |  | 0.97 | ( | 0.93 | - | 1.02 | ) | - | - |
|  | PC 16:1/22:6 | 0.99 | ( | 0.95 | - | 1.03 | ) | - | - |  | 0.97 | ( | 0.92 | - | 1.01 | ) | - | - |
|  | PC 18:0/18:1 | 1.00 | ( | 0.97 | - | 1.05 | ) | - | - |  | 1.00 | ( | 0.95 | - | 1.06 | ) | - | - |
|  | PC 18:0/18:2 | 1.00 | ( | 0.95 | - | 1.04 | ) | - | - |  | 1.07 | ( | 1.01 | - | 1.14 | ) | 0.017 | 0.008 |
|  | PC 18:0/20:3 | 0.98 | ( | 0.94 | - | 1.03 | ) | - | - |  | 1.01 | ( | 0.96 | - | 1.06 | ) | - | - |
|  | PC 18:0/20:4 | 1.02 | ( | 0.97 | - | 1.07 | ) | - | - |  | 1.12 | ( | 1.04 | - | 1.20 | ) | 0.004 | 0.002 |
|  | PC 18:0/20:5 | 1.01 | ( | 0.97 | - | 1.06 | ) | - | - |  | 1.02 | ( | 0.97 | - | 1.08 | ) | - | - |
|  | PC 18:0/22:5 | 1.02 | ( | 0.98 | - | 1.07 | ) | - | - |  | 0.96 | ( | 0.91 | - | 1.02 | ) | - | - |
|  | PC 18:0/22:6 | 1.01 | ( | 0.97 | - | 1.05 | ) | - | - |  | 1.00 | ( | 0.95 | - | 1.05 | ) | - | - |
|  | PC 18:1/18:2 | 1.00 | ( | 0.95 | - | 1.04 | ) | - | - |  | 1.09 | ( | 1.02 | - | 1.16 | ) | 0.007 | 0.004 |
|  | PC 18:1/22:6 | 0.99 | ( | 0.95 | - | 1.03 | ) | - | - |  | 1.00 | ( | 0.96 | - | 1.05 | ) | - | - |
|  | PC 18:2/20:4 | 0.99 | ( | 0.95 | - | 1.03 | ) | - | - |  | 0.98 | ( | 0.93 | - | 1.02 | ) | - | - |
|  | PC 20:0/18:2 | 1.01 | ( | 0.97 | - | 1.06 | ) | - | - |  | 1.00 | ( | 0.95 | - | 1.06 | ) | - | - |
|  | PC 20:4/20:4 | 0.98 | ( | 0.93 | - | 1.03 | ) | - | - |  | 0.95 | ( | 0.90 | - | 1.01 | ) | - | - |
|  | PC 20:4/22:6 | 0.98 | ( | 0.93 | - | 1.03 | ) | - | - |  | 0.98 | ( | 0.93 | - | 1.04 | ) | - | - |
| PE | PE 16:0/20:4 | 0.83 | ( | 0.76 | - | 0.91 | ) | <0.001 | <0.001 |  | 1.09 | ( | 1.02 | - | 1.15 | ) | 0.008 | 0.004 |
|  | PE 18:0/18:1 | 1.06 | ( | 1.01 | - | 1.10 | ) | 0.021 | 0.013 |  | 0.99 | ( | 0.93 | - | 1.05 | ) | - | - |
|  | PE 18:0/20:3 | 0.87 | ( | 0.81 | - | 0.93 | ) | <0.001 | <0.001 |  | 0.95 | ( | 0.90 | - | 1.01 | ) | - | - |
|  | PE 18:0/20:4 | 0.85 | ( | 0.78 | - | 0.92 | ) | <0.001 | <0.001 |  | 1.05 | ( | 0.99 | - | 1.10 | ) | - | - |
|  | PE 18:0/20:5 | 0.94 | ( | 0.89 | - | 0.98 | ) | 0.006 | 0.004 |  | 0.95 | ( | 0.91 | - | 1.00 | ) | - | - |
|  | PE 18:1/18:2 | 0.92 | ( | 0.87 | - | 0.97 | ) | 0.003 | 0.002 |  | 1.04 | ( | 0.98 | - | 1.11 | ) | - | - |
| PI | PI 16:0/20:4 | 1.06 | ( | 1.01 | - | 1.11 | ) | 0.025 | 0.015 |  | 0.91 | ( | 0.85 | - | 0.97 | ) | 0.006 | 0.003 |
|  | PI 18:0/18:1 | 1.06 | ( | 1.01 | - | 1.11 | ) | 0.016 | 0.010 |  | 0.83 | ( | 0.75 | - | 0.92 | ) | <0.001 | <0.001 |
|  | PI 18:0/18:2 | 1.04 | ( | 1.00 | - | 1.09 | ) | - | - |  | 0.94 | ( | 0.87 | - | 1.01 | ) | - | - |
|  | PI 18:0/20:3 | 1.07 | ( | 1.01 | - | 1.13 | ) | 0.018 | 0.011 |  | 0.93 | ( | 0.86 | - | 0.99 | ) | 0.033 | 0.014 |
|  | PI 18:0/20:4 | 1.05 | ( | 1.00 | - | 1.10 | ) | 0.035 | 0.020 |  | 0.89 | ( | 0.83 | - | 0.96 | ) | 0.004 | 0.002 |
|  | PI 18:0/22:5 | 1.07 | ( | 1.02 | - | 1.12 | ) | 0.003 | 0.002 |  | 0.92 | ( | 0.86 | - | 0.99 | ) | 0.026 | 0.012 |
|  | PI 18:0/22:6 | 1.06 | ( | 1.02 | - | 1.12 | ) | 0.009 | 0.006 |  | 0.96 | ( | 0.90 | - | 1.02 | ) | - | - |
|  | PI 18:1/18:2 | 1.06 | ( | 1.01 | - | 1.11 | ) | 0.022 | 0.013 |  | 0.87 | ( | 0.80 | - | 0.94 | ) | 0.001 | 0.001 |
|  | PI 18:1/20:4 | 1.08 | ( | 1.02 | - | 1.13 | ) | 0.005 | 0.003 |  | 0.87 | ( | 0.80 | - | 0.94 | ) | 0.001 | 0.001 |
| PC-o | PC o-16:0/18:2 | 0.97 | ( | 0.92 | - | 1.02 | ) | - | - |  | 1.03 | ( | 0.97 | - | 1.09 | ) | - | - |
|  | PC o-16:0/20:4 | 1.01 | ( | 0.96 | - | 1.06 | ) | - | - |  | 1.05 | ( | 0.99 | - | 1.11 | ) | - | - |
|  | PC o-16:0/22:6 | 1.01 | ( | 0.97 | - | 1.06 | ) | - | - |  | 1.02 | ( | 0.98 | - | 1.08 | ) | - | - |
|  | PC o-18:0/16:0 | 0.98 | ( | 0.93 | - | 1.03 | ) | - | - |  | 1.11 | ( | 1.04 | - | 1.18 | ) | 0.001 | 0.001 |
|  | PC o-18:0/18:2 | 1.00 | ( | 0.96 | - | 1.04 | ) | - | - |  | 1.03 | ( | 0.98 | - | 1.09 | ) | - | - |
|  | PC o-18:0/20:4 | 1.01 | ( | 0.97 | - | 1.06 | ) | - | - |  | 1.10 | ( | 1.03 | - | 1.17 | ) | 0.004 | 0.002 |
|  | PC o-18:0/22:6 | 1.02 | ( | 0.98 | - | 1.07 | ) | - | - |  | 1.04 | ( | 0.99 | - | 1.10 | ) | - | - |
|  | PC o-20:0/20:4 | 1.02 | ( | 0.97 | - | 1.07 | ) | - | - |  | 1.15 | ( | 1.07 | - | 1.23 | ) | <0.001 | <0.001 |
| PC-p | PC p-16:0/16:0 | 0.99 | ( | 0.94 | - | 1.03 | ) | - | - |  | 1.08 | ( | 1.02 | - | 1.13 | ) | 0.004 | 0.002 |
|  | PC p-16:0/20:4 | 0.97 | ( | 0.93 | - | 1.02 | ) | - | - |  | 1.06 | ( | 1.01 | - | 1.12 | ) | 0.016 | 0.008 |
|  | PC p-18:0/16:0 | 0.99 | ( | 0.94 | - | 1.04 | ) | - | - |  | 1.11 | ( | 1.05 | - | 1.18 | ) | <0.001 | <0.001 |
|  | PC p-18:0/18:1 | 1.01 | ( | 0.96 | - | 1.06 | ) | - | - |  | 1.08 | ( | 1.02 | - | 1.15 | ) | 0.005 | 0.003 |
|  | PC p-18:0/18:2 | 1.01 | ( | 0.96 | - | 1.05 | ) | - | - |  | 1.04 | ( | 0.99 | - | 1.10 | ) | - | - |
| PE-p | PE p-16:0/20:3 | 1.05 | ( | 1.00 | - | 1.09 | ) | - | - |  | 0.84 | ( | 0.76 | - | 0.93 | ) | 0.001 | 0.001 |
|  | PE p-16:0/20:4 | 1.03 | ( | 0.99 | - | 1.07 | ) | - | - |  | 0.86 | ( | 0.79 | - | 0.95 | ) | 0.002 | 0.001 |
|  | PE p-16:0/20:5 | 1.03 | ( | 0.99 | - | 1.08 | ) | - | - |  | 0.88 | ( | 0.81 | - | 0.96 | ) | 0.003 | 0.002 |
|  | PE p-16:0/22:6 | 1.05 | ( | 1.00 | - | 1.10 | ) | 0.040 | 0.022 |  | 0.92 | ( | 0.85 | - | 1.00 | ) | 0.043 | 0.018 |
|  | PE p-18:0/18:2 | 1.05 | ( | 1.00 | - | 1.10 | ) | 0.035 | 0.020 |  | 0.89 | ( | 0.82 | - | 0.97 | ) | 0.009 | 0.005 |
|  | PE p-18:0/20:4 | 1.05 | ( | 1.00 | - | 1.09 | ) | 0.033 | 0.019 |  | 0.86 | ( | 0.77 | - | 0.95 | ) | 0.004 | 0.002 |
|  | PE p-18:0/22:6 | 1.05 | ( | 1.00 | - | 1.09 | ) | 0.036 | 0.020 |  | 0.89 | ( | 0.82 | - | 0.98 | ) | 0.013 | 0.006 |
|  | PE p-18:1/20:4 | 1.04 | ( | 1.00 | - | 1.09 | ) | - | - |  | 0.85 | ( | 0.77 | - | 0.94 | ) | 0.002 | 0.001 |
|  | PE p-18:1/22:6 | 1.04 | ( | 0.99 | - | 1.09 | ) | - | - |  | 0.89 | ( | 0.81 | - | 0.98 | ) | 0.012 | 0.006 |
| DG | DG 16:0/18:1 | 0.76 | ( | 0.68 | - | 0.86 | ) | 0.000 | 0.000 |  | 0.97 | ( | 0.91 | - | 1.04 | ) | - | - |
|  | DG 18:1/18:1 | 1.07 | ( | 1.02 | - | 1.12 | ) | 0.008 | 0.005 |  | 1.17 | ( | 1.09 | - | 1.27 | ) | <0.001 | <0.001 |
|  | DG 18:1/18:2 | 1.06 | ( | 1.01 | - | 1.11 | ) | 0.017 | 0.011 |  | 1.17 | ( | 1.08 | - | 1.26 | ) | <0.001 | <0.001 |
| SM | SM d16:1/23:0 | 1.01 | ( | 0.96 | - | 1.07 | ) | - | - |  | 1.01 | ( | 0.96 | - | 1.07 | ) | - | - |
|  | SM d18:1/14:0 | 0.98 | ( | 0.93 | - | 1.03 | ) | - | - |  | 1.02 | ( | 0.97 | - | 1.07 | ) | - | - |
|  | SM d18:1/16:0 | 1.02 | ( | 0.97 | - | 1.08 | ) | - | - |  | 1.15 | ( | 1.07 | - | 1.23 | ) | <0.001 | <0.001 |
|  | SM d18:1/18:0 | 1.06 | ( | 0.99 | - | 1.14 | ) | - | - |  | 1.12 | ( | 1.04 | - | 1.20 | ) | 0.001 | 0.001 |
|  | SM d18:1/20:0 | 1.05 | ( | 0.99 | - | 1.11 | ) | - | - |  | 1.06 | ( | 1.00 | - | 1.12 | ) | 0.045 | 0.019 |
|  | SM d18:1/22:0 | 1.03 | ( | 0.97 | - | 1.09 | ) | - | - |  | 1.06 | ( | 1.00 | - | 1.13 | ) | - | - |
|  | SM d18:1/23:0 | 1.01 | ( | 0.95 | - | 1.07 | ) | - | - |  | 1.04 | ( | 0.98 | - | 1.10 | ) | - | - |
|  | SM d18:1/24:0 | 1.02 | ( | 0.96 | - | 1.07 | ) | - | - |  | 1.05 | ( | 0.99 | - | 1.11 | ) | - | - |
|  | SM d18:1/24:1 | 1.03 | ( | 0.98 | - | 1.08 | ) | - | - |  | 1.12 | ( | 1.05 | - | 1.19 | ) | <0.001 | <0.001 |
|  | SM d18:1/24:2 | 1.03 | ( | 0.98 | - | 1.08 | ) | - | - |  | 1.11 | ( | 1.05 | - | 1.18 | ) | <0.001 | <0.001 |
|  | SM d18:2/16:0 | 1.01 | ( | 0.95 | - | 1.07 | ) | - | - |  | 1.11 | ( | 1.04 | - | 1.18 | ) | 0.002 | 0.001 |
|  | SM d18:2/18:0 | 1.05 | ( | 0.99 | - | 1.13 | ) | - | - |  | 1.11 | ( | 1.04 | - | 1.19 | ) | 0.003 | 0.002 |
|  | SM d18:2/20:0 | 1.05 | ( | 1.00 | - | 1.11 | ) | 0.046 | 0.025 |  | 1.08 | ( | 1.02 | - | 1.14 | ) | 0.007 | 0.004 |
|  | SM d18:2/22:0 | 1.04 | ( | 0.99 | - | 1.09 | ) | - | - |  | 1.06 | ( | 1.01 | - | 1.13 | ) | 0.029 | 0.013 |
|  | SM d18:2/23:0 | 1.04 | ( | 0.99 | - | 1.09 | ) | - | - |  | 1.06 | ( | 1.00 | - | 1.13 | ) | 0.042 | 0.018 |
|  | SM d18:2/24:0 | 1.03 | ( | 0.98 | - | 1.08 | ) | - | - |  | 1.12 | ( | 1.05 | - | 1.19 | ) | <0.001 | <0.001 |
|  | SM d18:2/24:1 | 1.03 | ( | 0.99 | - | 1.08 | ) | - | - |  | 1.11 | ( | 1.05 | - | 1.18 | ) | 0.001 | 0.001 |
| Cer | Cer d18:0/24:1 | 1.06 | ( | 1.00 | - | 1.11 | ) | 0.036 | 0.020 |  | 1.16 | ( | 1.08 | - | 1.26 | ) | <0.001 | <0.001 |
|  | Cer d18:1/22:1 | 1.08 | ( | 1.02 | - | 1.14 | ) | 0.007 | 0.005 |  | 1.20 | ( | 1.10 | - | 1.32 | ) | <0.001 | <0.001 |
|  | Cer d18:1/24:1 | 1.03 | ( | 0.97 | - | 1.08 | ) | - | - |  | 1.16 | ( | 1.07 | - | 1.25 | ) | <0.001 | <0.001 |
| Glucer | Glucer d18:1/16:0 | 0.98 | ( | 0.93 | - | 1.04 | ) | - | - |  | 1.13 | ( | 1.06 | - | 1.21 | ) | <0.001 | <0.001 |
|  | Glucer d18:1/22:0 | 0.96 | ( | 0.92 | - | 1.01 | ) | - | - |  | 1.04 | ( | 1.00 | - | 1.09 | ) | - | - |
| CE | CE 18:2 | 1.00 | ( | 0.96 | - | 1.04 | ) | - | - |  | 1.04 | ( | 1.00 | - | 1.09 | ) | - | - |
|  | CE 18:3 | 1.04 | ( | 1.00 | - | 1.09 | ) | 0.036 | 0.020 |  | 1.07 | ( | 1.01 | - | 1.12 | ) | 0.011 | 0.006 |
|  | CE 20:3 | 1.04 | ( | 1.00 | - | 1.08 | ) | - | - |  | 1.04 | ( | 1.00 | - | 1.09 | ) | - | - |
|  | CE 20:4 | 1.06 | ( | 1.02 | - | 1.11 | ) | 0.008 | 0.005 |  | 1.07 | ( | 1.01 | - | 1.12 | ) | 0.011 | 0.006 |
|  | CE 20:5 | 1.03 | ( | 0.99 | - | 1.08 | ) | - | - |  | 1.07 | ( | 1.01 | - | 1.12 | ) | 0.014 | 0.007 |
|  | CE 22:6 | 1.13 | ( | 1.07 | - | 1.20 | ) | <0.001 | <0.001 |  | 1.09 | ( | 1.03 | - | 1.16 | ) | 0.006 | 0.003 |

OR, odds ratio; CI, confidence interval. Each p value was obtained from logistic regression model after adjustment for age, sex, BMI, LDL cholesterol, and fasting glucose. False discovery rate (FDR) q-value was calculated to correct for multiple comparisons.
