# Supplementary material for: Alteration in Metabolic Signature and Lipid Metabolism in Patients with Angina Pectoris and Myocardial Infarction
Source: PLoS One. 2015 Aug 10;10(8):e0135228. doi: 10.1371/journal.pone.0135228 (PMC4530944; doi:10.1371/journal.pone.0135228)
Supplement: S8 Table — (DOCX) [file pone.0135228.s009.docx]

| Metabolite | Control | | |  | Angina | | |  | MI | | | | | | |
| --- | --- | --- | --- | --- | --- | --- | --- | --- | --- | --- | --- | --- | --- | --- | --- |
|  | unadjusted | | |  | unadjusted | | |  | unadjusted | | |  | adjusted | | |
|  | r0 | p0 | q0 |  | r0 | p0 | q0 |  | r0 | p0 | q0 |  | r1 | p1 | q1 |
| PC 16:0/16:0 | -0.159 | - | - |  | 0.341 | 0.039 | - |  | 0.345 | <0.001 | 0.001 |  | 0.364 | <0.001 | 0.003 |
| PC 16:0/18:1 | -0.155 | - | - |  | -0.024 | - | - |  | 0.045 | - | - |  | 0.114 | - | - |
| PC 16:0/18:2 | -0.195 | - | - |  | 0.171 | - | - |  | 0.069 | - | - |  | 0.177 | - | - |
| PC 16:0/20:4 | -0.242 | - | - |  | -0.057 | - | - |  | 0.072 | - | - |  | 0.157 | - | - |
| PI 18:0/18:1 | -0.060 | - | - |  | -0.096 | - | - |  | -0.086 | - | - |  | 0.031 | - | - |
| PI 18:1/18:2 | -0.166 | - | - |  | -0.141 | - | - |  | -0.191 | - | - |  | -0.178 | - | - |
| PE p-16:0/20:3 | -0.117 | - | - |  | -0.110 | - | - |  | -0.289 | 0.030 | - |  | -0.153 | - | - |
| PE p-16:0/20:4 | -0.081 | - | - |  | -0.116 | - | - |  | -0.222 | - | - |  | -0.099 | - | - |
| PE p-16:0/20:5 | 0.012 | - | - |  | -0.049 | - | - |  | -0.225 | - | - |  | -0.103 | - | - |
| PE p-18:0/18:2 | -0.009 | - | - |  | -0.096 | - | - |  | -0.254 | - | - |  | -0.162 | - | - |
| PE p-18:0/20:4 | -0.050 | - | - |  | -0.190 | - | - |  | -0.176 | - | - |  | -0.042 | - | - |
| PE p-18:1/20:4 | -0.121 | - | - |  | -0.099 | - | - |  | -0.234 | - | - |  | -0.086 | - | - |
| PE p-18:1/22:6 | -0.079 | - | - |  | -0.033 | - | - |  | -0.148 | - | - |  | 0.026 | - | - |
| DG 18:1/18:2 | -0.256 | - | - |  | 0.113 | - | - |  | 0.404 | 0.002 | 0.006 |  | 0.377 | 0.007 | 0.020 |
| SM d18:1/16:0 | -0.223 | - | - |  | 0.181 | - | - |  | 0.392 | <0.001 | <0.001 |  | 0.438 | <0.001 | 0.001 |
| SM d18:1/18:0 | -0.168 | - | - |  | 0.188 | - | - |  | 0.443 | <0.001 | <0.001 |  | 0.487 | <0.001 | <0.001 |
| SM d18:1/24:1 | -0.070 | - | - |  | 0.208 | - | - |  | 0.313 | <0.001 | 0.002 |  | 0.363 | <0.001 | 0.003 |
| SM d18:1/24:2 | -0.073 | - | - |  | 0.145 | - | - |  | 0.326 | 0.001 | 0.004 |  | 0.279 | 0.009 | 0.023 |
| SM d18:2/18:0 | -0.118 | - | - |  | 0.065 | - | - |  | 0.377 | <0.001 | 0.001 |  | 0.403 | <0.001 | 0.002 |
| SM d18:2/24:0 | -0.090 | - | - |  | 0.186 | - | - |  | 0.290 | <0.001 | 0.002 |  | 0.362 | <0.001 | 0.002 |
| SM d18:2/24:1 | -0.089 | - | - |  | 0.114 | - | - |  | 0.327 | 0.001 | 0.003 |  | 0.287 | 0.006 | 0.019 |
| Cer d18:1/24:1 | -0.188 | - | - |  | 0.180 | - | - |  | 0.246 | 0.020 | - |  | 0.358 | 0.003 | 0.012 |
| Glucer d18:1/16:0 | -0.077 | - | - |  | 0.153 | - | - |  | 0.363 | <0.001 | <0.001 |  | 0.390 | <0.001 | 0.001 |

**S8 Table. Association of specific metabolites and the acute inflammatory response**

Unadjusted association was calculated to pearson’s correlation and presented with coefficients (r), p value (p0) and q value (q0). Adjusted association was calculated to partial correlation and presented coefficients (r1), p value (p1) and q value (q1) controlled for age, sex, BMI, LDL cholesterol and fasting glucose.
