# Supplementary material for: Alteration in Metabolic Signature and Lipid Metabolism in Patients with Angina Pectoris and Myocardial Infarction
Source: PLoS One. 2015 Aug 10;10(8):e0135228. doi: 10.1371/journal.pone.0135228 (PMC4530944; doi:10.1371/journal.pone.0135228)
Supplement: S9 Table — (DOCX) [file pone.0135228.s010.docx]

**S9 Table. Levels of individual lipid species in patients with angina and MI according to statin treatment**

| Class | Metabolites | Angina | | | | | | |  | MI | | | | | | |
| --- | --- | --- | --- | --- | --- | --- | --- | --- | --- | --- | --- | --- | --- | --- | --- | --- |
|  |  | Non-treatment | | | Treatment | | | P value |  | Non-treatment | | | Treatment | | | P value |
| FFA | FFA 16:0 | 479.3 | ± | 39.9 | 466.9 | ± | 23.1 | - |  | 275.5 | ± | 21.0 | 281.6 | ± | 15.2 | - |
|  | FFA 16:1 | 143.8 | ± | 10.9 | 125.3 | ± | 8.2 | - |  | 70.9 | ± | 7.2 | 73.0 | ± | 5.2 | - |
|  | FFA 18:0 | 211.4 | ± | 16.4 | 217.3 | ± | 10.4 | - |  | 131.6 | ± | 9.4 | 134.7 | ± | 6.5 | - |
|  | FFA 18:1 | 687.2 | ± | 56.6 | 650.9 | ± | 34.2 | - |  | 371.8 | ± | 30.6 | 381.0 | ± | 21.8 | - |
|  | FFA 18:2 | 453.1 | ± | 42.0 | 409.8 | ± | 24.3 | - |  | 223.1 | ± | 19.0 | 233.2 | ± | 16.8 | - |
|  | FFA 18:3 | 103.0 | ± | 9.5 | 88.7 | ± | 7.8 | - |  | 32.0 | ± | 3.6 | 40.9 | ± | 4.8 | - |
|  | FFA 20:1 | 19.0 | ± | 2.8 | 15.6 | ± | 1.2 | - |  | 9.8 | ± | 1.3 | 9.9 | ± | 1.1 | - |
|  | FFA 20:2 | 16.3 | ± | 1.7 | 13.8 | ± | 1.0 | - |  | 6.7 | ± | 0.7 | 7.1 | ± | 0.6 | - |
|  | FFA 20:3 | 19.9 | ± | 1.8 | 18.1 | ± | 1.5 | - |  | 7.6 | ± | 0.9 | 8.2 | ± | 0.8 | - |
|  | FFA 20:4 | 44.9 | ± | 4.0 | 46.4 | ± | 3.8 | - |  | 22.7 | ± | 2.5 | 23.0 | ± | 1.6 | - |
|  | FFA 20:5 | 22.2 | ± | 2.7 | 26.5 | ± | 3.5 | - |  | 7.6 | ± | 1.1 | 8.6 | ± | 1.3 | - |
|  | FFA 22:3 | 0.4 | ± | 0.0 | 0.4 | ± | 0.0 | - |  | 0.2 | ± | 0.0 | 0.2 | ± | 0.0 | - |
|  | FFA 22:6 | 114.1 | ± | 13.1 | 107.5 | ± | 9.7 | - |  | 40.5 | ± | 5.3 | 46.1 | ± | 6.1 | - |
|  | FFA 24:5 | 3.7 | ± | 0.5 | 3.0 | ± | 0.4 | - |  | 1.0 | ± | 0.2 | 1.3 | ± | 0.2 | - |
|  | FFA 24:6 | 2.0 | ± | 0.2 | 2.4 | ± | 0.3 | - |  | 0.6 | ± | 0.1 | 0.7 | ± | 0.1 | - |
| LysoPC | LysoPC 14:0 | 3.1 | ± | 0.2 | 3.1 | ± | 0.1 | - |  | 2.6 | ± | 0.2 | 2.7 | ± | 0.1 | - |
|  | LysoPC 16:0 | 303.9 | ± | 9.0 | 292.4 | ± | 7.9 | - |  | 320.0 | ± | 17.6 | 322.6 | ± | 12.2 | - |
|  | LysoPC 16:1 | 9.5 | ± | 0.5 | 9.1 | ± | 0.3 | - |  | 8.8 | ± | 0.4 | 9.1 | ± | 0.4 | - |
|  | LysoPC 18:0 | 125.2 | ± | 4.2 | 120.5 | ± | 4.1 | - |  | 118.4 | ± | 7.8 | 122.3 | ± | 5.6 | - |
|  | LysoPC 18:1 | 76.3 | ± | 3.1 | 69.2 | ± | 2.2 | - |  | 63.5 | ± | 4.1 | 63.6 | ± | 2.7 | - |
|  | LysoPC 18:2 | 131.9 | ± | 9.1 | 107.3 | ± | 5.1 | 0.039 |  | 82.8 | ± | 7.0 | 90.8 | ± | 7.0 | - |
|  | LysoPC 18:3 | 3.4 | ± | 0.3 | 3.0 | ± | 0.2 | - |  | 2.1 | ± | 0.2 | 2.3 | ± | 0.2 | - |
|  | LysoPC 18:4 | 1.4 | ± | 0.1 | 1.3 | ± | 0.0 | - |  | 1.2 | ± | 0.1 | 1.3 | ± | 0.0 | - |
|  | LysoPC 20:1 | 2.3 | ± | 0.1 | 2.1 | ± | 0.1 | - |  | 2.1 | ± | 0.1 | 2.1 | ± | 0.1 | - |
|  | LysoPC 20:3 | 16.1 | ± | 1.3 | 14.6 | ± | 0.9 | - |  | 11.0 | ± | 0.8 | 12.2 | ± | 0.9 | - |
|  | LysoPC 20:4 | 31.3 | ± | 2.7 | 32.1 | ± | 2.0 | - |  | 28.8 | ± | 2.3 | 29.1 | ± | 2.0 | - |
|  | LysoPC 20:5 | 8.4 | ± | 0.6 | 9.7 | ± | 0.7 | - |  | 5.3 | ± | 0.5 | 6.2 | ± | 0.6 | - |
|  | LysoPC 22:5 | 5.2 | ± | 0.3 | 4.9 | ± | 0.4 | - |  | 3.0 | ± | 0.3 | 3.3 | ± | 0.3 | - |
|  | LysoPC 22:6 | 35.7 | ± | 2.5 | 32.4 | ± | 2.2 | - |  | 20.0 | ± | 2.1 | 22.7 | ± | 2.0 | - |
|  | LysoPC 24:0 | 0.6 | ± | 0.0 | 0.6 | ± | 0.0 | - |  | 0.6 | ± | 0.0 | 0.6 | ± | 0.0 | - |
| LysoPE | LysoPE 16:0 | 1.9 | ± | 0.1 | 1.9 | ± | 0.1 | - |  | 2.2 | ± | 0.2 | 2.2 | ± | 0.1 | - |
|  | LysoPE 18:0 | 3.2 | ± | 0.2 | 2.9 | ± | 0.1 | - |  | 3.6 | ± | 0.3 | 3.6 | ± | 0.1 | - |
|  | LysoPE 18:1 | 0.9 | ± | 0.0 | 1.0 | ± | 0.0 | - |  | 1.0 | ± | 0.0 | 1.0 | ± | 0.0 | - |
|  | LysoPE 18:2 | 6.0 | ± | 0.6 | 4.6 | ± | 0.3 | - |  | 4.7 | ± | 0.5 | 5.4 | ± | 0.5 | - |
|  | LysoPE 18:3 | 0.2 | ± | 0.0 | 0.2 | ± | 0.0 | - |  | 0.3 | ± | 0.0 | 0.3 | ± | 0.0 | - |
|  | LysoPE 20:1 | 1.9 | ± | 0.0 | 2.0 | ± | 0.0 | - |  | 1.8 | ± | 0.0 | 1.8 | ± | 0.0 | - |
|  | LysoPE 20:3 | 1.7 | ± | 0.1 | 1.6 | ± | 0.1 | - |  | 1.6 | ± | 0.1 | 1.7 | ± | 0.1 | - |
|  | LysoPE 20:4 | 6.4 | ± | 0.8 | 5.6 | ± | 0.3 | - |  | 6.0 | ± | 0.7 | 6.3 | ± | 0.5 | - |
|  | LysoPE 22:1 | 1.1 | ± | 0.0 | 1.1 | ± | 0.0 | - |  | 1.1 | ± | 0.0 | 1.1 | ± | 0.0 | - |
|  | LysoPE 22:5 | 0.4 | ± | 0.1 | 0.4 | ± | 0.0 | - |  | 0.4 | ± | 0.0 | 0.5 | ± | 0.0 | - |
|  | LysoPE 22:6 | 11.4 | ± | 1.2 | 10.4 | ± | 0.6 | - |  | 10.8 | ± | 1.2 | 12.1 | ± | 1.1 | - |
| LysoPC-o | LysoPC o-16:0 | 5.0 | ± | 0.2 | 5.0 | ± | 0.1 | - |  | 5.2 | ± | 0.2 | 5.2 | ± | 0.2 | - |
|  | LysoPC o-18:0 | 6.8 | ± | 0.3 | 7.0 | ± | 0.3 | - |  | 6.3 | ± | 0.4 | 6.2 | ± | 0.3 | - |
| LysoPC-p | LysoPC p-18:0 | 2.8 | ± | 0.1 | 2.7 | ± | 0.1 | - |  | 3.0 | ± | 0.2 | 2.9 | ± | 0.1 | - |
| PC | PC 14:0/18:2 | 15.5 | ± | 0.8 | 12.5 | ± | 0.6 | 0.010 |  | 12.7 | ± | 0.8 | 13.1 | ± | 0.7 | - |
|  | PC 14:0/20:5 | 1.0 | ± | 0.1 | 1.1 | ± | 0.1 | - |  | 0.7 | ± | 0.1 | 0.8 | ± | 0.1 | - |
|  | PC 16:0/16:0 | 50.7 | ± | 1.7 | 45.8 | ± | 1.1 | - |  | 63.5 | ± | 2.2 | 59.7 | ± | 1.6 | - |
|  | PC 16:0/16:1 | 48.5 | ± | 3.6 | 41.1 | ± | 2.4 | - |  | 53.5 | ± | 3.4 | 53.5 | ± | 2.8 | - |
|  | PC 16:0/18:0 | 12.9 | ± | 0.4 | 11.6 | ± | 0.3 | - |  | 13.8 | ± | 0.6 | 12.9 | ± | 0.4 | - |
|  | PC 16:0/18:1 | 633.7 | ± | 24.5 | 552.9 | ± | 15.7 | - |  | 733.6 | ± | 30.2 | 712.8 | ± | 20.0 | - |
|  | PC 16:0/18:2 | 1207.0 | ± | 50.0 | 1048.6 | ± | 36.5 | 0.027 |  | 1342.4 | ± | 51.8 | 1351.4 | ± | 40.8 | - |
|  | PC 16:0/18:3 | 15.1 | ± | 0.8 | 12.5 | ± | 0.7 | - |  | 14.2 | ± | 1.0 | 13.9 | ± | 0.8 | - |
|  | PC 16:0/20:3 | 365.8 | ± | 17.1 | 321.6 | ± | 12.4 | - |  | 381.2 | ± | 15.3 | 397.3 | ± | 13.1 | - |
|  | PC 16:0/20:4 | 454.4 | ± | 29.9 | 464.5 | ± | 18.3 | - |  | 609.3 | ± | 39.0 | 603.2 | ± | 28.3 | - |
|  | PC 16:0/20:5 | 208.9 | ± | 17.2 | 236.0 | ± | 16.0 | - |  | 204.6 | ± | 21.0 | 219.6 | ± | 15.8 | - |
|  | PC 16:0/22:4 | 27.8 | ± | 1.8 | 24.6 | ± | 1.2 | - |  | 26.9 | ± | 1.8 | 28.6 | ± | 1.9 | - |
|  | PC 16:0/22:5 | 121.1 | ± | 6.2 | 118.3 | ± | 4.2 | - |  | 127.3 | ± | 7.3 | 127.3 | ± | 6.4 | - |
|  | PC 16:0/22:6 | 605.9 | ± | 27.6 | 571.2 | ± | 21.4 | - |  | 656.0 | ± | 40.3 | 673.1 | ± | 24.4 | - |
|  | PC 16:1/18:2 | 58.9 | ± | 3.8 | 48.0 | ± | 2.6 | 0.015 |  | 52.8 | ± | 2.6 | 58.1 | ± | 3.6 | - |
|  | PC 16:1/20:4 | 13.7 | ± | 1.0 | 11.5 | ± | 0.4 | 0.030 |  | 11.3 | ± | 0.7 | 12.3 | ± | 0.8 | - |
|  | PC 16:1/22:6 | 14.1 | ± | 0.5 | 13.7 | ± | 0.4 | - |  | 12.1 | ± | 0.5 | 13.0 | ± | 0.5 | - |
|  | PC 18:0/18:1 | 140.6 | ± | 7.6 | 122.1 | ± | 5.5 | - |  | 132.7 | ± | 7.0 | 132.1 | ± | 4.5 | - |
|  | PC 18:0/18:2 | 687.3 | ± | 29.7 | 578.1 | ± | 21.4 | 0.022 |  | 659.2 | ± | 20.9 | 698.7 | ± | 25.1 | - |
|  | PC 18:0/20:3 | 125.2 | ± | 8.2 | 119.8 | ± | 6.4 | - |  | 123.8 | ± | 7.9 | 134.4 | ± | 7.3 | - |
|  | PC 18:0/20:4 | 248.2 | ± | 18.5 | 260.9 | ± | 11.8 | - |  | 299.0 | ± | 15.5 | 304.8 | ± | 14.7 | - |
|  | PC 18:0/20:5 | 0.6 | ± | 0.0 | 0.6 | ± | 0.0 | - |  | 0.6 | ± | 0.0 | 0.6 | ± | 0.0 | - |
|  | PC 18:0/22:5 | 34.7 | ± | 2.1 | 35.7 | ± | 2.3 | - |  | 28.5 | ± | 1.6 | 31.7 | ± | 1.9 | - |
|  | PC 18:0/22:6 | 222.3 | ± | 8.5 | 222.4 | ± | 10.7 | - |  | 214.5 | ± | 11.1 | 232.7 | ± | 9.5 | - |
|  | PC 18:1/18:2 | 203.6 | ± | 10.4 | 179.9 | ± | 6.4 | - |  | 220.9 | ± | 9.8 | 220.5 | ± | 7.7 | - |
|  | PC 18:1/22:6 | 29.2 | ± | 1.6 | 25.6 | ± | 1.0 | - |  | 26.1 | ± | 1.2 | 27.3 | ± | 1.0 | - |
|  | PC 18:2/20:4 | 36.5 | ± | 1.9 | 34.8 | ± | 1.3 | - |  | 32.0 | ± | 2.2 | 32.5 | ± | 1.7 | - |
|  | PC 20:0/18:2 | 22.2 | ± | 1.0 | 18.0 | ± | 0.6 | 0.006 |  | 18.8 | ± | 0.6 | 20.1 | ± | 0.6 | - |
|  | PC 20:4/20:4 | 6.1 | ± | 0.3 | 5.4 | ± | 0.2 | - |  | 5.3 | ± | 0.3 | 5.4 | ± | 0.3 | - |
|  | PC 20:4/22:6 | 1.8 | ± | 0.1 | 1.8 | ± | 0.1 | - |  | 1.8 | ± | 0.1 | 1.8 | ± | 0.1 | - |
| PE | PE 16:0/20:4 | 4.1 | ± | 0.3 | 4.0 | ± | 0.2 | - |  | 7.4 | ± | 0.7 | 6.9 | ± | 0.3 | - |
|  | PE 18:0/18:1 | 2.2 | ± | 0.2 | 2.1 | ± | 0.1 | - |  | 2.0 | ± | 0.2 | 1.8 | ± | 0.1 | - |
|  | PE 18:0/20:3 | 0.6 | ± | 0.0 | 0.7 | ± | 0.0 | - |  | 0.7 | ± | 0.1 | 0.8 | ± | 0.0 | - |
|  | PE 18:0/20:4 | 6.5 | ± | 0.6 | 6.4 | ± | 0.3 | - |  | 10.4 | ± | 1.0 | 10.2 | ± | 0.5 | - |
|  | PE 18:0/20:5 | 1.3 | ± | 0.1 | 1.6 | ± | 0.1 | - |  | 1.4 | ± | 0.1 | 1.6 | ± | 0.1 | - |
|  | PE 18:1/18:2 | 0.8 | ± | 0.1 | 0.7 | ± | 0.0 | - |  | 1.0 | ± | 0.1 | 1.0 | ± | 0.1 | - |
| PI | PI 16:0/20:4 | 5.5 | ± | 0.4 | 5.8 | ± | 0.3 | - |  | 4.1 | ± | 0.4 | 4.1 | ± | 0.2 | - |
|  | PI 18:0/18:1 | 3.6 | ± | 0.3 | 3.5 | ± | 0.2 | - |  | 2.3 | ± | 0.2 | 2.3 | ± | 0.1 | - |
|  | PI 18:0/18:2 | 26.5 | ± | 2.6 | 22.3 | ± | 1.2 | - |  | 17.4 | ± | 1.3 | 18.1 | ± | 0.9 | - |
|  | PI 18:0/20:3 | 8.0 | ± | 0.7 | 7.6 | ± | 0.4 | - |  | 4.6 | ± | 0.4 | 5.5 | ± | 0.3 | 0.010 |
|  | PI 18:0/20:4 | 57.8 | ± | 4.2 | 59.6 | ± | 2.3 | - |  | 41.6 | ± | 3.1 | 43.8 | ± | 1.8 | 0.038 |
|  | PI 18:0/22:5 | 2.5 | ± | 0.2 | 2.8 | ± | 0.1 | 0.036 |  | 1.7 | ± | 0.2 | 1.8 | ± | 0.1 | - |
|  | PI 18:0/22:6 | 10.3 | ± | 1.1 | 9.4 | ± | 0.7 | - |  | 6.8 | ± | 1.1 | 7.2 | ± | 0.5 | - |
|  | PI 18:1/18:2 | 2.5 | ± | 0.2 | 2.2 | ± | 0.1 | - |  | 1.4 | ± | 0.1 | 1.5 | ± | 0.1 | - |
|  | PI 18:1/20:4 | 2.2 | ± | 0.2 | 2.2 | ± | 0.1 | - |  | 1.5 | ± | 0.1 | 1.5 | ± | 0.1 | - |
| PC-o | PC o-16:0/18:2 | 18.3 | ± | 1.2 | 15.1 | ± | 0.7 | - |  | 19.7 | ± | 0.9 | 18.5 | ± | 0.8 | - |
|  | PC o-16:0/20:4 | 56.7 | ± | 3.3 | 56.3 | ± | 2.4 | - |  | 63.5 | ± | 2.9 | 60.8 | ± | 2.4 | - |
|  | PC o-16:0/22:6 | 23.2 | ± | 1.2 | 22.9 | ± | 0.8 | - |  | 24.9 | ± | 1.3 | 23.9 | ± | 0.9 | - |
|  | PC o-18:0/16:0 | 1.0 | ± | 0.0 | 0.8 | ± | 0.0 | - |  | 1.1 | ± | 0.0 | 1.1 | ± | 0.0 | - |
|  | PC o-18:0/18:2 | 39.7 | ± | 2.3 | 35.2 | ± | 1.3 | - |  | 37.6 | ± | 1.7 | 38.6 | ± | 1.5 | - |
|  | PC o-18:0/20:4 | 13.7 | ± | 0.8 | 13.5 | ± | 0.8 | - |  | 16.0 | ± | 0.8 | 15.1 | ± | 0.6 | - |
|  | PC o-18:0/22:6 | 7.0 | ± | 0.4 | 6.9 | ± | 0.2 | - |  | 7.2 | ± | 0.3 | 7.1 | ± | 0.3 | - |
|  | PC o-20:0/20:4 | 2.8 | ± | 0.1 | 2.7 | ± | 0.1 | - |  | 3.5 | ± | 0.2 | 3.1 | ± | 0.1 | - |
| PC-p | PC p-16:0/16:0 | 6.7 | ± | 0.3 | 5.8 | ± | 0.2 | - |  | 8.0 | ± | 0.4 | 7.2 | ± | 0.3 | - |
|  | PC p-16:0/20:4 | 34.4 | ± | 1.9 | 33.7 | ± | 1.3 | - |  | 44.5 | ± | 2.2 | 39.0 | ± | 1.2 | 0.021 |
|  | PC p-18:0/16:0 | 19.0 | ± | 0.7 | 16.8 | ± | 0.5 | - |  | 22.6 | ± | 0.9 | 21.3 | ± | 0.8 | - |
|  | PC p-18:0/18:1 | 51.6 | ± | 2.8 | 47.8 | ± | 1.6 | - |  | 57.9 | ± | 2.4 | 55.0 | ± | 2.0 | - |
|  | PC p-18:0/18:2 | 13.2 | ± | 0.8 | 12.0 | ± | 0.5 | - |  | 13.5 | ± | 0.6 | 13.0 | ± | 0.5 | - |
| PE-p | PE p-16:0/20:3 | 4.6 | ± | 0.5 | 4.5 | ± | 0.3 | - |  | 2.7 | ± | 0.3 | 2.5 | ± | 0.2 | - |
|  | PE p-16:0/20:4 | 8.5 | ± | 0.8 | 9.5 | ± | 0.7 | - |  | 6.0 | ± | 0.7 | 5.1 | ± | 0.3 | - |
|  | PE p-16:0/20:5 | 4.7 | ± | 0.6 | 5.5 | ± | 0.6 | - |  | 2.6 | ± | 0.6 | 2.3 | ± | 0.3 | - |
|  | PE p-16:0/22:6 | 12.2 | ± | 1.2 | 13.0 | ± | 1.0 | - |  | 8.5 | ± | 1.0 | 8.2 | ± | 0.5 | - |
|  | PE p-18:0/18:2 | 4.3 | ± | 0.4 | 3.9 | ± | 0.3 | - |  | 2.2 | ± | 0.3 | 2.3 | ± | 0.2 | - |
|  | PE p-18:0/20:4 | 13.0 | ± | 1.3 | 15.4 | ± | 1.3 | - |  | 8.8 | ± | 1.1 | 8.1 | ± | 0.6 | - |
|  | PE p-18:0/22:6 | 9.2 | ± | 0.9 | 9.9 | ± | 0.7 | - |  | 5.7 | ± | 0.8 | 5.6 | ± | 0.4 | - |
|  | PE p-18:1/20:4 | 11.8 | ± | 1.2 | 13.0 | ± | 0.9 | - |  | 7.6 | ± | 0.9 | 7.0 | ± | 0.4 | - |
|  | PE p-18:1/22:6 | 5.4 | ± | 0.6 | 5.7 | ± | 0.5 | - |  | 3.4 | ± | 0.4 | 3.3 | ± | 0.2 | - |
| DG | DG 16:0/18:1 | 5.7 | ± | 0.6 | 4.9 | ± | 0.3 | - |  | 7.4 | ± | 0.6 | 8.4 | ± | 0.5 | - |
|  | DG 18:1/18:1 | 400.9 | ± | 16.1 | 409.3 | ± | 10.7 | - |  | 432.0 | ± | 11.7 | 444.5 | ± | 11.2 | - |
|  | DG 18:1/18:2 | 119.6 | ± | 4.9 | 122.7 | ± | 3.4 | - |  | 128.9 | ± | 3.7 | 133.1 | ± | 3.5 | - |
| SM | SM d16:1/23:0 | 14.9 | ± | 0.7 | 14.0 | ± | 0.5 | - |  | 15.3 | ± | 0.8 | 15.0 | ± | 0.7 | - |
|  | SM d18:1/14:0 | 34.6 | ± | 1.3 | 32.2 | ± | 1.1 | - |  | 39.0 | ± | 1.4 | 36.8 | ± | 1.6 | - |
|  | SM d18:1/16:0 | 316.0 | ± | 12.3 | 284.3 | ± | 8.2 | - |  | 366.4 | ± | 11.6 | 349.3 | ± | 11.0 | - |
|  | SM d18:1/18:0 | 56.3 | ± | 2.6 | 50.5 | ± | 1.5 | - |  | 67.5 | ± | 3.7 | 67.2 | ± | 3.2 | - |
|  | SM d18:1/20:0 | 40.9 | ± | 2.0 | 38.2 | ± | 1.2 | - |  | 43.8 | ± | 2.0 | 43.4 | ± | 2.0 | - |
|  | SM d18:1/22:0 | 93.8 | ± | 5.3 | 82.5 | ± | 3.0 | - |  | 98.1 | ± | 4.8 | 98.7 | ± | 5.0 | - |
|  | SM d18:1/23:0 | 40.4 | ± | 1.9 | 36.8 | ± | 1.3 | - |  | 42.4 | ± | 2.2 | 43.5 | ± | 1.9 | - |
|  | SM d18:1/24:0 | 70.6 | ± | 3.2 | 64.6 | ± | 2.4 | - |  | 75.4 | ± | 3.9 | 76.7 | ± | 3.8 | - |
|  | SM d18:1/24:1 | 45.4 | ± | 1.9 | 42.3 | ± | 1.2 | - |  | 53.0 | ± | 1.7 | 51.6 | ± | 1.7 | - |
|  | SM d18:1/24:2 | 18.7 | ± | 0.9 | 17.6 | ± | 0.7 | - |  | 22.5 | ± | 1.0 | 21.5 | ± | 0.8 | - |
|  | SM d18:2/16:0 | 59.4 | ± | 2.5 | 53.1 | ± | 1.8 | - |  | 69.1 | ± | 2.8 | 66.4 | ± | 2.2 | - |
|  | SM d18:2/18:0 | 32.8 | ± | 1.7 | 29.9 | ± | 1.1 | - |  | 38.5 | ± | 2.1 | 38.1 | ± | 1.6 | - |
|  | SM d18:2/20:0 | 18.4 | ± | 0.8 | 17.9 | ± | 0.5 | - |  | 20.3 | ± | 0.7 | 20.0 | ± | 0.7 | - |
|  | SM d18:2/22:0 | 61.8 | ± | 2.6 | 57.6 | ± | 1.8 | - |  | 65.5 | ± | 2.1 | 64.3 | ± | 2.2 | - |
|  | SM d18:2/23:0 | 30.6 | ± | 1.3 | 29.5 | ± | 0.9 | - |  | 33.4 | ± | 1.1 | 32.4 | ± | 1.0 | - |
|  | SM d18:2/24:0 | 206.8 | ± | 9.3 | 190.3 | ± | 5.4 | - |  | 246.1 | ± | 9.3 | 235.6 | ± | 8.4 | - |
|  | SM d18:2/24:1 | 84.9 | ± | 3.9 | 79.9 | ± | 2.6 | - |  | 101.1 | ± | 4.2 | 96.0 | ± | 3.3 | - |
| Cer | Cer d18:0/24:1 | 22.2 | ± | 0.8 | 22.3 | ± | 0.6 | - |  | 24.0 | ± | 0.7 | 24.4 | ± | 0.7 | - |
|  | Cer d18:1/22:1 | 1.5 | ± | 0.1 | 1.5 | ± | 0.0 | - |  | 1.5 | ± | 0.0 | 1.7 | ± | 0.0 | - |
|  | Cer d18:1/24:1 | 7.5 | ± | 0.4 | 6.9 | ± | 0.2 | - |  | 8.6 | ± | 0.3 | 8.2 | ± | 0.2 | - |
| Glucer | Glucer d18:1/16:0 | 0.8 | ± | 0.0 | 0.7 | ± | 0.0 | - |  | 1.1 | ± | 0.0 | 0.9 | ± | 0.0 | - |
|  | Glucer d18:1/22:0 | 1.4 | ± | 0.1 | 1.2 | ± | 0.1 | - |  | 1.6 | ± | 0.1 | 1.5 | ± | 0.1 | - |
| CE | CE 18:2 | 8.6 | ± | 0.5 | 8.4 | ± | 0.3 | - |  | 8.9 | ± | 0.4 | 9.4 | ± | 0.4 | - |
|  | CE 18:3 | 2.2 | ± | 0.1 | 2.3 | ± | 0.1 | - |  | 2.2 | ± | 0.1 | 2.3 | ± | 0.1 | - |
|  | CE 20:3 | 0.7 | ± | 0.0 | 0.7 | ± | 0.0 | - |  | 0.7 | ± | 0.0 | 0.8 | ± | 0.0 | - |
|  | CE 20:4 | 6.0 | ± | 0.4 | 7.1 | ± | 0.3 | - |  | 7.1 | ± | 0.4 | 6.9 | ± | 0.3 | - |
|  | CE 20:5 | 22.1 | ± | 1.5 | 21.7 | ± | 1.0 | - |  | 21.3 | ± | 1.0 | 23.8 | ± | 1.0 | - |
|  | CE 22:6 | 5.4 | ± | 0.3 | 6.2 | ± | 0.3 | - |  | 5.1 | ± | 0.3 | 5.2 | ± | 0.2 | - |

The data are presented as the mean ± SE. Each p value was obtained from the general linear model after adjustment for age, sex, BMI, and LDL cholesterol and fasting glucose. Q values of all metabolites were not significant (q>0.05).
