# Supplementary material for: Alteration in Metabolic Signature and Lipid Metabolism in Patients with Angina Pectoris and Myocardial Infarction
Source: PLoS One. 2015 Aug 10;10(8):e0135228. doi: 10.1371/journal.pone.0135228 (PMC4530944; doi:10.1371/journal.pone.0135228)
Supplement: S10 Table — (DOCX) [file pone.0135228.s011.docx]

**S10 Table. ORs for CAD of individual lipid species associated with statin treatment**

| Disease | Metabolites | Non-treatment | | | | | | | |  | Treatment | | | | | | | |
| --- | --- | --- | --- | --- | --- | --- | --- | --- | --- | --- | --- | --- | --- | --- | --- | --- | --- | --- |
|  |  | OR | (95%CI) | | | | | p value | q value |  | OR | (95%CI) | | | | | p value | q value |
| Angina | LysoPC 18:2 | 1.39 | ( | 1.19 | - | 1.61 | ) | <0.001 | <0.001 |  | 1.25 | ( | 1.13 | - | 1.40 | ) | <0.001 | <0.001 |
|  | PC 14:0/18:2 | 1.00 | ( | 0.95 | - | 1.06 | ) | - |  |  | 0.89 | ( | 0.82 | - | 0.96 | ) | 0.002 | 0.002 |
|  | PC 16:0/18:2 | 1.07 | ( | 1.00 | - | 1.15 | ) | - |  |  | 0.96 | ( | 0.90 | - | 1.03 | ) | - |  |
|  | PC 16:1/18:2 | 1.03 | ( | 0.98 | - | 1.08 | ) | - |  |  | 0.94 | ( | 0.88 | - | 1.00 | ) | - |  |
|  | PC 16:1/20:4 | 1.02 | ( | 0.97 | - | 1.07 | ) | - |  |  | 0.94 | ( | 0.88 | - | 1.00 | ) | - |  |
|  | PC 18:0/18:2 | 1.06 | ( | 0.99 | - | 1.13 | ) | - |  |  | 0.96 | ( | 0.90 | - | 1.02 | ) | - |  |
|  | PC 20:0/18:2 | 1.07 | ( | 1.01 | - | 1.13 | ) | 0.021 | 0.022 |  | 0.96 | ( | 0.90 | - | 1.02 | ) | - |  |
|  | PI 18:0/22:5 | 1.05 | ( | 0.99 | - | 1.11 | ) | - |  |  | 1.10 | ( | 1.04 | - | 1.17 | ) | 0.001 | 0.001 |
|  | PC p-16:0/20:4 | 0.97 | ( | 0.92 | - | 1.03 | ) | - |  |  | 1.03 | ( | 0.97 | - | 1.10 | ) | - |  |
| MI | PI 18:0/20:3 | 0.73 | ( | 0.60 | - | 0.89 | ) | 0.002 | 0.006 |  | 0.97 | ( | 0.90 | - | 1.05 | ) | - |  |
|  | PI 18:0/20:4 | 0.74 | ( | 0.60 | - | 0.92 | ) | 0.006 | 0.009 |  | 0.93 | ( | 0.86 | - | 1.01 | ) | - |  |
|  | PC p-16:0/20:4 | 1.12 | ( | 1.04 | - | 1.21 | ) | 0.004 | 0.006 |  | 1.05 | ( | 0.98 | - | 1.11 | ) | - |  |

OR, odds ratio; CI, confidence interval.

Each P-value was obtained from the logistic regression model after adjusting for age, sex, BMI, LDL cholesterol and fasting glucose. False discovery rate (FDR) q-value was calculated to correct for multiple comparisons.
